# Regulation of Catalase Expression and Activity by *Dh*Hog1 in the Halotolerant Yeast *Debaryomyces hansenii* Under Saline and Oxidative Conditions

**DOI:** 10.3390/jof10110740

**Published:** 2024-10-26

**Authors:** Ileana de la Fuente-Colmenares, James González, Norma Silvia Sánchez, Daniel Ochoa-Gutiérrez, Viviana Escobar-Sánchez, Claudia Segal-Kischinevzky

**Affiliations:** 1 Laboratorio de Biología Molecular y Genómica, Departamento de Biología Celular, Facultad de Ciencias, Universidad Nacional Autónoma de México, Avenida Universidad # 3000, Cd. Universitaria, Coyoacán, Mexico City 04510, Mexico; ile.delafc@ciencias.unam.mx (I.d.l.F.-C.); biolibre@ciencias.unam.mx (D.O.-G.); vv.escobar@ciencias.unam.mx (V.E.-S.); 2 Posgrado en Ciencias Biológicas, Universidad Nacional Autónoma de México, Avenida Universidad # 3000, Cd. Universitaria, Coyoacán, Mexico City 04510, Mexico; 3 Departamento de Genética Molecular, Instituto de Fisiología Celular, Universidad Nacional Autónoma de México, Ciudad Universitaria, Mexico City 04510, Mexico; nsanchez@ifc.unam.mx

**Keywords:** transcriptional regulation, stress response, catalase induction, stress conditions, HOG pathway, HOG MAPK

## Abstract

Efficient transcriptional regulation of the stress response is critical for microorganism survival. In yeast, stress-related gene expression, particularly for antioxidant enzymes like catalases, mitigates reactive oxygen species such as hydrogen peroxide (H_2_O_2_), preventing cell damage. The halotolerant yeast *Debaryomyces hansenii* shows oxidative stress tolerance, largely due to high catalase activity from *DhCTA* and *DhCTT* genes. This study evaluates *D. hansenii’*s response to oxidative stress caused by H_2_O_2_ under saline conditions, focusing on cell viability, gene expression, and catalase activity. Chromatin organization in the promoter of *DhCTA* and *DhCTT* was analyzed, revealing low nucleosome occupancy in promoter regions, correlating with active gene expression. Stress-related motifs for transcription factors like Msn2/4 and Sko1 were found, suggesting regulation by the *DhHog1* MAP kinase. Analysis of a *Dhhog1*Δ mutant showed *Dh*Hog1’s role in *DhCTA* expression under H_2_O_2_ or NaCl conditions. These findings highlight *Dh*Hog1’s critical role in regulating the stress response in *D. hansenii*, offering insights for enhancing stress tolerance in halotolerant yeasts, particularly for industrial applications in saline wastewater management.

## 1. Introduction

**Figure 1:**
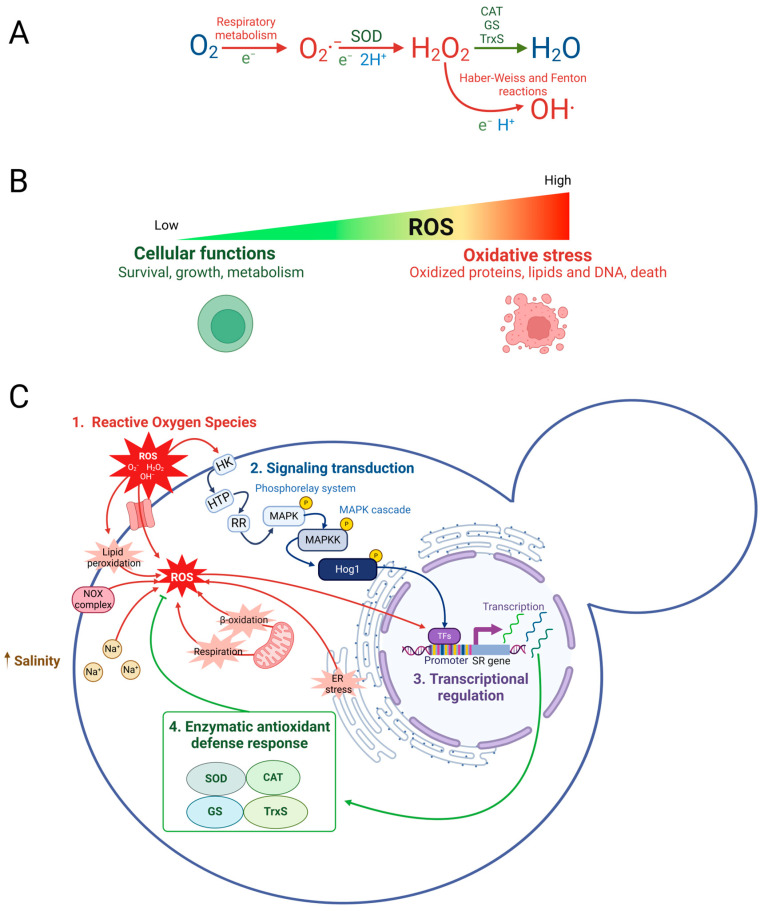
Schematic representation of reactive oxygen species (ROS) triggering oxidative stress response. (**A**) ROS, such as superoxide anion (O_2_·^−^), are produced during oxygen metabolism and converted to hydrogen peroxide (H_2_O_2_) by superoxide dismutase (SOD). H_2_O_2_ is further broken down by catalase (CAT), the glutathione system (GS), and the thioredoxin system (TrxS). (**B**) Low ROS levels maintain homeostasis, while elevated levels cause oxidative stress, damaging lipids, proteins, and DNA, leading to cell death. (**C**) Oxidative stress response: (i) ROS sources (red) include NADPH oxidases, lipid peroxidation, respiration, β-oxidation, endoplasmic reticulum (ER) stress or external factors such as high salinity (Na+). ROS can disturb redox homeostasis, resulting in oxidative damage. (ii) Signal transduction (blue) involves a phosphorelay system (hybrid sensor histidine kinase HK, phosphoryl transfer protein HPT) and a response regulator protein (RR). This system activates the Hog1 MAP kinase cascade (MAPK and MAPKK), leading to the phosphorylation and activation of Hog1. (iii) Phosphorylated Hog1 modulates transcription factors (purple) like Msn2/4 and Sko1 to regulate stress response genes (SR genes). (iv) Enzymatic antioxidant defense (green, SOD, CAT, GS, TrxS) neutralize ROS. Based on [20,23,24]. Created using BioRender.com with license UY27G222GA.

In yeast, exposure to moderate oxidative stress conditions triggers a transient adaptive response [1], enhancing tolerance to higher levels of the same stress [2,3]. Salt and oxidative stress tolerance is acquired through osmolyte synthesis and increased scavenging of ROS [4,5,6]. Oxidative stress primarily arises from internal metabolic processes, particularly respiration, as well as external stressors [7,8,9]. ROS, such as superoxide anions (O_2_·^−^), hydrogen peroxide (H_2_O_2_), and hydroxyl radicals (·OH), are inevitable by-products of aerobic metabolism (Figure 1A). While ROS are crucial for cellular signaling and homeostasis maintenance, their excessive accumulation can overwhelm the antioxidant defenses of the cell, leading to oxidative stress (Figure 1B). This condition occurs when the balance between ROS production and elimination is disturbed, potentially causing cytotoxic effects, such as lipid, protein, and nucleic acid damage, which can result in cell death [7,8,10].

To mitigate the deleterious effects of ROS, aerobic microorganisms have evolved sophisticated antioxidant defense mechanisms, which are particularly crucial in yeast under oxidative stress (Figure 1C) [11,12]. The cellular stress response encompasses a suite of defense mechanisms activated in response to oxidative stress, involving several transcription factors (TFs) such as Msn2/Msn4 (Msn2/4), Sko1, Skn7, Yap1, among others, to regulate the expression of hundreds of genes aimed at countering stress and repairing cellular damage [13,14,15,16,17]. The oxidative stress response is characterized by the upregulation of genes encoding antioxidant enzymes, such as catalases (CAT), which play pivotal roles in scavenging ROS. CAT catalyzes the breakdown of H_2_O_2_ into H_2_O and O_2_, thereby preventing the harmful accumulation of ROS [11,18,19,20]. The expression of CAT is tightly regulated at the transcriptional level, often involving chromatin remodeling mechanisms that either facilitate or inhibit gene expression in response to oxidative stress [21,22].

The yeast *Saccharomyces cerevisiae* is useful as a model for studying oxidative stress responses, where the high osmolarity glycerol (HOG) MAP kinase pathway plays a central role, which consists of a phosphorylation cascade that activates *Sc*Hog1 MAPK (mitogen-activated protein kinase) [25,26,27,28]. The phosphorylated *Sc*Hog1 protein is essential for activating TFs (Figure 1C), such as Msn2/4 and Sko1, which are critical for the expression of antioxidant genes [20,29]. The *Schog1*Δ mutant significantly reduces the expression of these genes, and its cell viability decreases under stress conditions, highlighting its importance in the oxidative stress response [30]. *Debaryomyces hansenii*, a halotolerant yeast, provides a unique model for studying stress responses due to its ability to thrive in hyperosmotic environments [31,32,33]. These conditions typically induce cellular stress, disturbing redox homeostasis and leading to oxidative damage. Its halotolerance is attributed to the robustness of the hyperosmolarity response of the HOG pathway [34,35]. It has been reported that the phosphorylated Hog1 protein directs the adaptive response for cell survival through glycerol accumulation and reprogramming the expression of genes that mitigate osmotic stress [36]. Unlike *S. cerevisiae*, *D. hansenii* displays significantly higher catalase activity, attributed to the expression of two catalase genes, *DhCTA* and *DhCTT*, homologous to *ScCTA* and *ScCTT* in *S. cerevisiae* [11,37]. These catalase genes are constitutively expressed under various growth conditions, including saline conditions, suggesting that *D. hansenii* has evolved a robust antioxidant defense system to cope with its challenging osmotic environments [11,38].

Although the role of the *Dh*Hog1 pathway in *D. hansenii* halotolerance is well established [34,35], its specific contribution to the regulation of catalase expression under oxidative conditions has not yet been evaluated. Given the crucial function of catalases in mitigating ROS-induced damage [11,39], it is essential to understand the mechanisms governing the expression of *DhCTA* and *DhCTT* under oxidative stress. This study aimed to investigate the adaptive response of *D. hansenii* by analyzing catalase expression and activity under saline and oxidative conditions. Our findings indicate that exposure to H_2_O_2_ shock reduces cell viability while transiently increasing catalase expression and activity. Additionally, low nucleosome occupancy was observed in the promoters of *DhCTA* and *DhCTT*, correlating with their active expression. Finally, *Dh*Hog1 was identified as a key regulator of *DhCTA* expression under both saline and oxidative conditions.

## 2. Material and Methods

### 2.1. Strains and Growth Conditions

The *D. hansenii* Y7426 wild-type (WT) and an isogenic *Dhhog1*Δ mutant (*HOG1*::*SAT1*-yeYFP1), obtained by [35], were used in this study. The WT strain was maintained on YPD (1% yeast extract, 2% peptone, and 2% glucose, *w*/*v*) plates with 2% agar (*w*/*v*), while the *Dhhog*1Δ mutant strain was maintained on YPD plates with 2% agar (*w*/*v*) supplemented with 150 μg/mL nourseothricin.

Cultures were pre-grown overnight, washed, and inoculated into fresh YPD medium with or without 0.6 M NaCl, as specified in each assay, to an initial optical density at 600 nm (OD_600_) of 0.05. Cells were then incubated until the mid-exponential growth phase (14–16 h). Cultures were incubated at 28 °C with continuous shaking (180 rpm).

### 2.2. Growth Curves

WT and *Dhhog1*Δ strains were grown in YPD with and without 0.6 M NaCl. Cell growth was monitored by measuring OD_600_. Cultures in the corresponding growth media were inoculated at an OD_600_ of 0.05 with water-washed cells from the pre-culture and monitored for 72 h. Each growth curve was performed in triplicate.

### 2.3. Cell Viability After H_2_O_2_ Gradient Shock Assay

Cells were inoculated in 5 mL aliquots of YPD with 0.6 M NaCl and different concentrations of H_2_O_2_ (0, 2.5, 5, 10, 20, and 30 mM), adjusting the OD_600_ to 1. The cultures were incubated for 3 h. After the oxidative treatment, H_2_O_2_ was removed by washing the cells once with sterile water. The washed cells were resuspended in 5 mL of sterile water and tenfold serially diluted. From each dilution, 10 μL were spotted on YPD plates and incubated at 28 °C for 3 or more days.

H_2_O_2_ sensitivity assays at different time intervals were evaluated following a modified method from [40]. Cells were harvested by centrifugation, washed with sterile water, and adjusted to an OD_600_ of 1 in fresh YPD with 0.6 M NaCl supplemented with 30 mM H_2_O_2_. The cultures were incubated for 180 min, with aliquots taken at 0, 15, 30, 60, 120, and 180 min and also spotted on YPD plates and incubated at 28 °C; photographs were taken after 3 or more days.

### 2.4. Specific Catalase Activity Determination

Cell-free crude extracts were prepared from 50–100 mL aliquots of culture to test specific catalase activity. Cells were lysed in PBS (pH 7) 100 mM + 20% glycerol using sterile glass microbeads (425–600 μm, Sigma G9268, St. Louis, MO, USA) vortexing for 1 min and incubating on ice for 1 min, and the procedure was repeated 4 times. After cold centrifugation for 15 min at 14,000 rpm, protein extracts were kept on ice until the specific catalase activity assay, which was conducted using varying volumes of the extract (1, 2.5, 5, 10, 20 or 40 μL, total volume 1 mL) and protein quantification with the Bradford assay, using a BSA protein standard curve, were performed.

Specific catalase activity was determined by a method adapted from [41]. A sample (1–40 μL) of the cell-free crude extract was mixed with 2.9 mL of assay mixture (100 mM sodium phosphate buffer, pH 7.0, and 1 μL/100 mL Triton X-100) in a 3 mL quartz cuvette. The reaction was initiated by adding 100 μL of 500 mM H_2_O_2_ (final concentration of 16.6 mM). Catalase activity was monitored by OD_240nm_ decay over 3 min. Catalase total activity was calculated based on the rate of decomposition of H_2_O_2_, which is inversely proportional to the reduction of absorbance at 240 nm. Catalase activities were normalized to total protein and expressed as µmol of H_2_O_2_ oxidized per minute per mg of protein.

### 2.5. RNA Extraction

Strains were grown in YPD with or without 0.6 M NaCl to the mid-log growth phase, and then fresh media was inoculated to have samples of 0, 14, or 24 h. Cells were incubated with or without 30 mM H_2_O_2_ for 0, 15, 30, 60, 120, and 180 min. After treatment, cells were washed with sterile water to remove the H_2_O_2_. Total yeast RNA was extracted from 50–100 mL of cell cultures following a modified method from [42]. Briefly, cells were washed with sterile DEPC-treated water, resuspended in AE buffer (50 mM sodium acetate, 10 mM EDTA), and mechanically disrupted using a vortex mixer with sterile glass microbeads (425–600 μm, Sigma G9268), phenol pH 4.5 and 0.25% SDS. Then, the samples were incubated for 5 min at 65 °C and vortexed for 30 s twice. The mixture was chilled and centrifuged at 16,000× *g* to separate the aqueous and phenol phases. The aqueous phase was extracted twice with phenol:chloroform:isoamyl alcohol (25:24:1) and once with chloroform:isoamyl alcohol (24:1). RNA precipitation was performed by adding 0.1 volumes of 3 M sodium acetate and 2.5 volumes of cold absolute ethanol and incubated at −20 °C for 30 min, followed by centrifugation (16,000× *g*). The resulting pellet was washed in 75% ethanol, air-dried, and resuspended in RNase-free water. RNA integrity was verified with 1% denaturing agarose gel electrophoresis, and concentration was determined by measuring A260/280 and A260/230 ratios using a nanospectrophotometer (Perl IMPLEN, Implen GmbH, München, Germany).

### 2.6. Analysis of Gene Expression

Total RNA was digested with DNAse I (Q1 RNase-Free DNase, Promega, Madison, WI, USA) to remove any contaminating genomic DNA, and cDNA synthesis was performed using the RevertAid H Minus First Strand cDNA Synthesis kit (Thermo Scientific, Waltham, MA, USA) following the manufacturer’s recommendations using random primers. RT-qPCR analysis was performed using primers initially screened for the absence of dimer formation and cross-hybridization. Primer pairs with 90–100% amplification efficiencies were used (Table 1). The qPCR analysis was conducted using a Rotor-Gene Q machine from Qiagen (Hilden, Germany) and the KAPA SYBR FAST (BIOSYSTEMS, Cape Town, South Africa) as a detector dye, following the specified profile settings: 95 °C for 3 min (1 cycle), 95 °C for 15 s, 58 °C for 15 s, and 72 °C for 15 s (30 cycles), followed by a final extension at 72 °C for 10 min. Transcripts of *DhCTA* (ID: 2904338, locus DEHA2F10582g), *DhCTT* (ID: 2913632, locus DEHA2B16214g) and *DhSTL1* (ID: 2902951, locus DEHA2E01364g) were normalized using transcripts of *DhRPS3* (ID: 2905489, locus DEHA2G22770g) [43] or *DhACT1* (ID: 2901278, locus DEHA2D05412g) [35] by the standard curve method. The mean value ± SD of three biological replicates is presented. Fold change was calculated by normalizing the H_2_O_2_-treated sample’s relative expression against non-treated ones (YPD).

### 2.7. Nucleosome Scanning Assay

Nucleosome scanning assays (NuSA) were performed by adapting a previously described method [44,45]. Cells were grown at mid-log in YPD with 0.6 M NaCl and compared against cells grown in YPD with 0.6 M NaCl incubated with 30 mM H_2_O_2_ for 60 min. Briefly, cells were cross-linked with 1% formaldehyde and incubated for 20 min at room temperature. Subsequently, cells were treated with 125 mM glycine and incubated for 5 min at room temperature. Samples were then centrifuged and washed with Tris-buffered saline (50 mM Tris-Cl, pH 7.5, 150 mM NaCl), followed by incubation in Buffer Z2 (1 M sorbitol, 50 mM Tris-Cl at pH 7.4, 10 mM β-mercaptoethanol) containing 2 mg of zymolyase-20T (Sigma L2524) for 20 min at 30 °C on a shaker. Spheroplasts were pelleted by centrifugation at 3000 rpm and resuspended in 1.5 mL of NPS buffer (0.5 mM spermidine, 0.075% NP-40, 50 mM NaCl, 10 mM Tris, pH 7.4, 5 mM MgCl_2_, 1 mM CaCl_2_, 1 mM β-mercaptoethanol).

DNA digestions with MNase were performed as previously reported [46]. Samples were divided into three 500-µL aliquots, which were then digested with 22.5 units of MNase (Nuclease S7 from Roche) from 10 to 50 min at 37 °C. Digestions were stopped with 12 μL of stop buffer (50 mM EDTA and 1% SDS) and treated with 100 µg of proteinase K at 65 °C overnight. DNA was extracted twice with phenol/chloroform and precipitated with 20 µL of 5 M NaCl (100 mM) and 685 µL of isopropanol (1:1) overnight at −20 °C. The precipitates were resuspended in 40 µL of TE buffer and incubated with 20 mg RNase A for 1 h at 37 °C. Samples with monosomal bands were cut and purified using the GFX PCR DNA and Gel Band Purification Kit (reference 28903470; Illustra, Buckinghamshire, UK).

DNA samples were diluted 1:20 and used for qPCR to independently determine the relative MNase protection of *DhCTA* (DEHA2F10582g) and *DhCTT* (DEHA2B16214g) promoter regions (−625 to +250 bp). qPCR was carried out as follows: 95 °C for 5 min (1 cycle), 95 °C for 15 s, 58 °C for 20 s, and 72 °C for 20 s (30 cycles). The relative protection of *DhCTT* and *DhCTA* was calculated as a ratio considering the amplification of a region of *DhVCX1-2* with the following deoxyoligonucleotides pair: forward 5′-TCTCCAGTCAATTACCTTTTGG-3′; and reverse, 5′-AATTCAACCAGAAAATGGCATTAG-3′ (Figure S2E,F). PCR deoxyoligonucleotides for *DhCTT* and *DhCTA* promoters are described in Tables S2 and S3, which amplify from around −625 to +250 bp of each locus, with coordinates given relative to the ATG +1. All presented NuSAs represent the mean values and standard errors (SE) of at least two independent biological replicates and four technical replicates.

### 2.8. Promoter Analysis

The nucleotide sequences of the genes *DhCTA* (ID: 2904338, locus DEHA2F10582g), *DhCTT* (ID: 2913632, locus DEHA2B16214g), *DhSOD1* (ID: 2905248, locus DEHA2G17732g), *DhENA1* (ID: 2904382, locus DEHA2G09108g), *DhSTL1* (ID: 2902951, locus DEHA2E01364g), and *DhGPD1* (ID: 2903610, locus DEHA2F09372g) were obtained from the NCBI Gene database (https://www.ncbi.nlm.nih.gov/gene/) accessed on 3 September 2023. An intergenic region spanning from −625 bp upstream to the start codon of each gene was analyzed using the Regulatory Sequence Analysis Tools (RSAT) platform (https://rsat.france-bioinformatique.fr/fungi/) accessed on 11 November 2023, specifically employing the matrix-scan quick tool within the Fungi Server (https://rsat.france-bioinformatique.fr/fungi/matrix-scan_form.cgi) accessed on 11 November 2023 [47]. Binding motifs to transcription factors associated with stress response were identified, including *MSN2/4* (ID: 2899670, locus DEHA2A08382g), *SKN7* (ID: 2913769, locus DEHA2B08052g), *SKO1* (ID: 2901375, locus DEHA2D09196g), and *YAP1* (ID: 904514, locus DEHA2G02420g). These transcription factors were selected based on the presence of predicted orthologs in the *D. hansenii* genome, identified through the predicted ortholog cluster from the Candida Gene Order Browser [48] or from the list of orthologs in *D. hansenii* previously identified by the best reciprocal hit method against *S. cerevisiae* genes [49]. The matrices for each motif were retrieved from the JASPAR database: *MSN2/4* (ID: MA0341.1/MA0342.1), *SKN7* (ID: MA038.1), *SKO1* (ID: MA0382.1), and *YAP1* (ID: MA0415.1), using the 2020 core non-redundant fungi collection. An organism-specific background model estimation method was applied, and a *p*-value threshold of E3 was used for significant determination.

### 2.9. Identification of Putative Orthologs of Transcription Factors

The amino acid sequences of transcription factor proteins were retrieved from the NCBI protein database (https://www.ncbi.nlm.nih.gov/protein accessed on 6 July 2024) for the following: *Sc*Msn2/4 (ID: NP_013751.1/NP_012861.1), *Sc*Skn7 (ID: KZV10951.1), *Sc*Sko1 (ID: NP_014232.1), and *Sc*Yap1 (ID: KZV08838.1) from *S. cerevisiae*; *Dh*Msn2 (ID: CAG84649.2), *Dh*Skn7 (ID: CAG85310.2), *Dh*Sko1 (ID: XP_458864.2), and *Dh*Yap1 (ID: XP_461648.2) from *D. hansenii*; and *Ca*Msn4 (ID: XP_723438.2), *Ca*Skn7 (ID: AAQ08008.1), *Ca*Sko1 (ID: XP_019330633.1), and Cap1 (ID: KAL1577880.1) from *C. albicans*. The groups of orthologous TFs were aligned using Clustal Omega v 1.2.4 (EMBL-EBI, Hinxton, UK) on 9 July 2024 [50]. The overall percentage identity and conserved domains between the orthologs of *S. cerevisiae*, *D. hansenii*, and *C. albicans* were calculated using the pairwise alignment tool in Jalview 2.11.3 [51].

### 2.10. Statistical Analysis

Statistical significance was assessed using two-way ANOVA, followed by Tukey’s multiple comparison test, or Brown-Forsythe and Welsch ANOVA tests, followed by post hoc multiple comparison according to Dunnett, using GraphPad Prism v 8.4.2 software. All experiments were conducted with a minimum of three biological replicates. Statistically significant differences are indicated as follows: **** *p* ≤ 0.0001; *** *p* < 0.001; ** *p* < 0.01; * *p* < 0.05.

## 3. Results

### 3.1. H_2_O_2_-Induced Oxidative Stress Triggers Catalase Expression and Activity in D. hansenii

**Figure 2:**
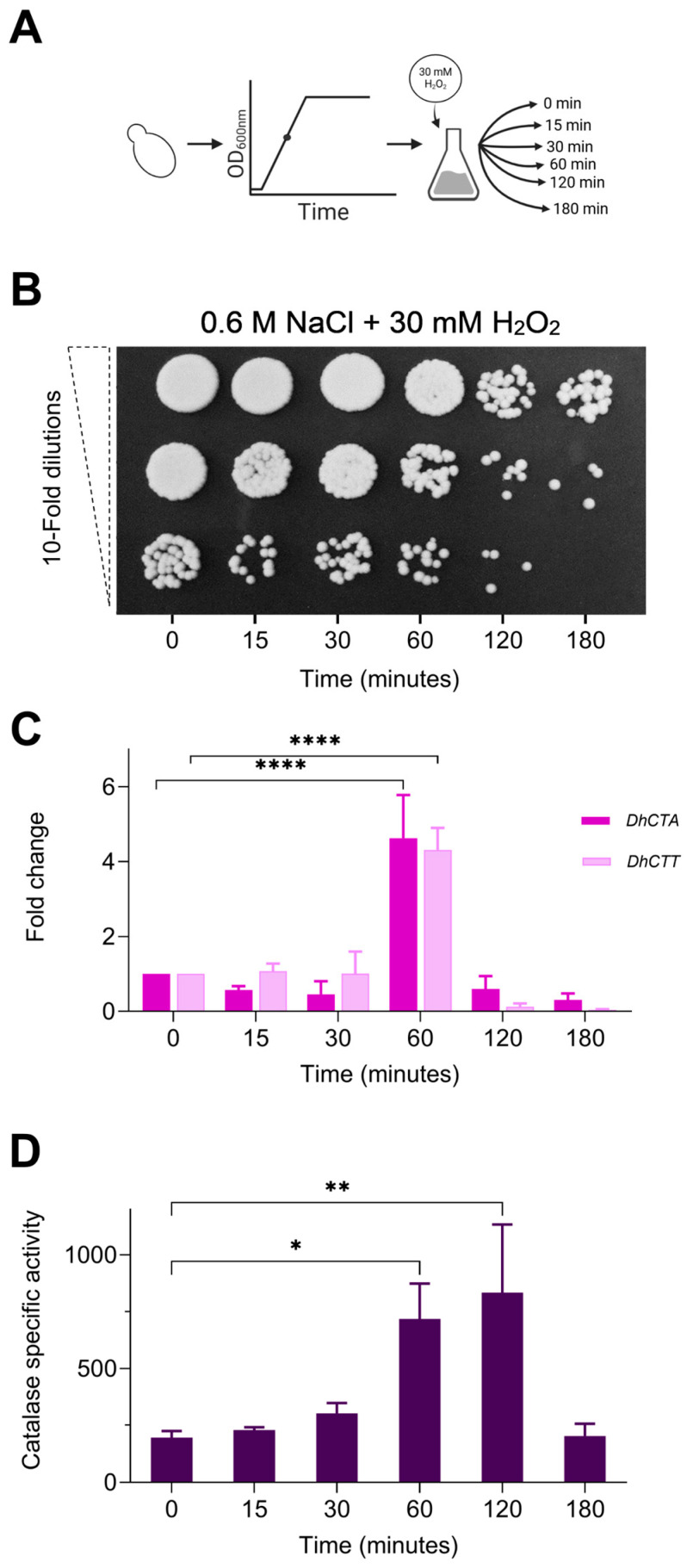
Time course response to hydrogen peroxide exposure, measuring cellular viability, *DhCTA* and *DhCTT* expression, and catalase-specific enzyme activity. (**A**) Cells were grown in YPD with 0.6 M NaCl to the mid-log phase, as described in the Materials and Methods section. (**B**) Assessment of cell survival following H_2_O_2_ shock (30 mM) at different time intervals (0, 15, 30, 60, 120, and 180 min) with shaking, followed by tenfold serial dilutions (10^−2^, 10^−3^, 10^−4^). A 10-μL aliquot of each dilution was spotted onto YPD agar plates and incubated for 3 days at 28 °C. (**C**) Relative expression (fold change) of the *DhCTA* (dark bars) and *DhCTT* (light bars) genes. Total RNA was extracted from cells at different intervals and analyzed by RT-qPCR. Transcript data were normalized against the expression level of the ribosomal protein S3 gene (*DhRPS3*). (**D**) Catalase activity in cell-free yeast extracts was measured at different intervals (μmol H_2_O_2_ oxidized min^−1^ mg of protein^−1^). Values are presented as the mean of at least six independent measurements ± (SD). Significant differences: *p*-value ≤ 0.05 (*), ≤0.01 (**), ≤0.0001 (****).

*D. hansenii* shows the capacity to tolerate various environmental stressors, including salt and oxidative stress [31,52]. To assess oxidative stress tolerance in *D. hansenii* under saline conditions, cells were grown in rich YPD medium supplemented with 0.6 M NaCl until reaching an OD_600_ of 1, followed by exposure to a hydrogen peroxide (H_2_O_2_) shock across a concentration gradient (0–50 mM) for 3 h. Serial dilutions and spot assays were subsequently performed (Figure S1), as detailed in the Materials and Methods section. Significant sensitivity was observed at 30 mM H_2_O_2_, leading to the selection of this concentration for subsequent experiments (Figure 2A). Aliquots were collected to analyze cell viability at different time intervals (0, 15, 30, 60, 120, and 180 min), revealing a decrease in viability after 60 min (Figure 2B). Subsequently, the expression and activity of catalase A (*Dh*Cta) and catalase T (*Dh*Ctt) were analyzed in cells treated with 30 mM H_2_O_2_ with 0.6 M NaCl as previously described (Figure 2A). RT-qPCR analysis showed a transient increase of up to 5-fold in the expression of both *DhCTA* and *DhCTT* (Figure 2C). This upregulation in gene expression was accompanied by a corresponding increase in specific catalase activity, which peaked between 60 and 120 min after H_2_O_2_ exposure before returning to baseline levels by the third hour of treatment (Figure 2D). Notably, basal catalase activity is moderately high in the absence of H_2_O_2_. These findings suggest that *D. hansenii* can tolerate oxidative stress induced by H_2_O_2_ under saline conditions, responding with a transient upregulation of *DhCTA* and *DhCTT* catalase expression and activity.

### 3.2. Nucleosome Occupancy and Putative TF Binding Motifs Distribution in DhCTA and DhCTT Promoters

The expression of catalase genes in *S. cerevisiae* is regulated through a complex interplay of mechanisms, including oxidative stress responses, general stress signals, nutrient availability, and epigenetic regulation [53]. This multifaceted regulation allows the yeast to precisely adjust its antioxidant response according to environmental conditions and the physiological state of the cell, thereby ensuring its survival and adaptation in changing environments.

**Figure 3:**
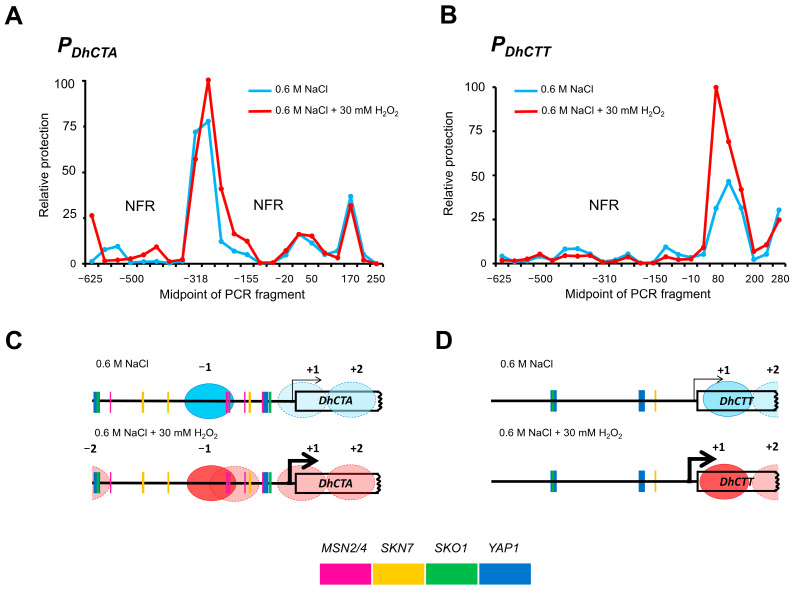
Nucleosome scanning assay indicates that *DhCTA* and *DhCTT* expression induction is independent of chromatin, which remains accessible under NaCl conditions or NaCl plus H_2_O_2_ shock conditions. (**A**,**B**) Relative MNase protection of *DhCTA* and *DhCTT* genes was calculated as the ratio of template present in MNase-digested DNA to the amount of MNase protection observed for the *DhVCX1* locus, which was used as control. NFR stands for Nucleosome-Free Regions. (**C**,**D**) Diagrams of each locus indicate the positions of nucleosomes (circles) extrapolated from the MNase protection data. The thickness of the arrows indicates transcript levels under each condition. Colored boxes correspond to the binding sites of *MSN2/4*, *SKN7*, *SKO1,* and *YAP1* in each promoter (*P_DhCTA_* and *P_DhCTT_*).

**Figure 4:**
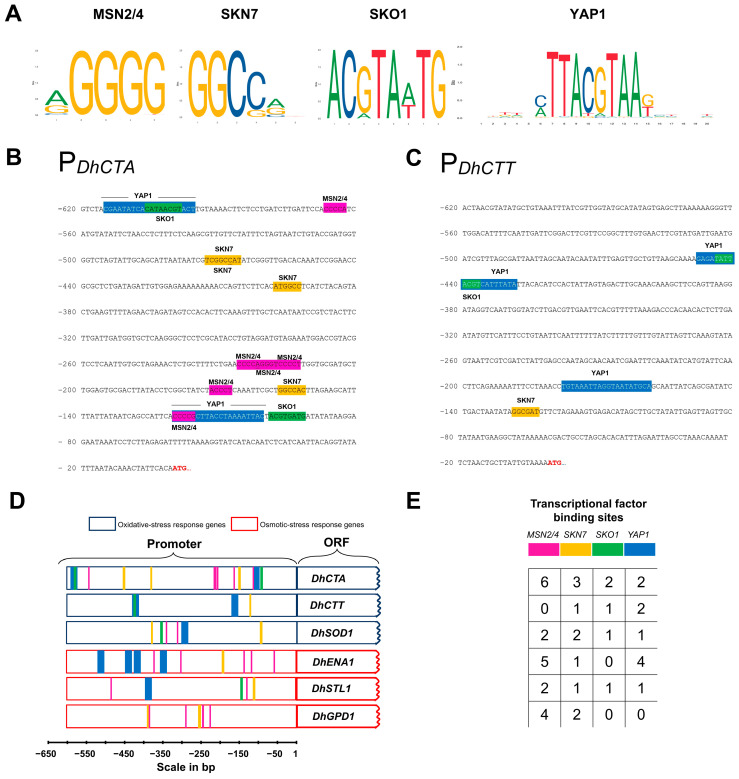
Predicted stress-related TF binding sites in *DhCTA*, *DhCTT*, *DhSOD1*, *DhENA1*, *DhSTL1*, and *DhGPD1* promoter regions. (**A**) Sequence logos defining *MSN2/4*, *SKN7*, *SKO1*, and *YAP1* are shown as provided by the Jaspar database http://jaspar.genereg.net/ accessed on 11 November 2023. (**B**,**C**) Sequences from −620 bp upstream to +1 ATG are shown; the predicted motifs are highlighted for *MSN2/4* (pink), *SKN7* (yellow), *SKO1* (green), and *YAP1* (blue). (**B**) For *DhCTA*, (**C**) For *DhCTT*. (**D**) Oxidative and saline stress-related genes (dark blue outlines), osmotic-stress response genes (red outlines). (**E**) Number of TF binding sites identified in each promoter.

To investigate the regulation of catalases in *D. hansenii*, we analyzed chromatin arrangement under NaCl exposure at an initial time point and after 60 min of combined NaCl and H_2_O_2_ shock, during which an increase in transcript levels was observed (Figure 2C). This analysis was conducted using a Nucleosome Scanning Assay (NuSA) targeting the proximal promoters of both genes. To our knowledge, this study represents the first mononucleosome isolation and nucleosome scanning report in *D. hansenii* (Figure S2). The analysis extended up to −625 bp from the proximal promoter of both loci, revealing that nucleosome occupancy under these conditions is minimal (Figure 3A,B), a characteristic feature of highly active genes. Furthermore, both genes exhibited Nucleosome-Free Regions (NFR), and the nucleosome positioning remained consistent across the two conditions analyzed. The *DhCTA* gene exhibited the −2, −1, +1, and +2 nucleosomes, while *DhCTT* showed the +1 and +2 nucleosomes. Remarkably, nucleosome occupancy did not change in response to H_2_O_2_ shock in both genes. However, the −1 nucleosome is strongly positioned in the *DhCTA* promoter in both conditions (Figure 3C), while the +1 nucleosome is more fixed in the *DhCTT* promoter after the H_2_O_2_ (45% vs. 100%, relative protection) (Figure 3D). To further understand the regulation of these genes, an in silico analysis was conducted to identify putative TF binding motifs within both promoters (Figure 4B,C). In the *DhCTA* promoter, we identified motifs for *MSN2/4*, *SKN7*, *SKO1*, and *YAP1* in the NFR (Figure 4B). Under oxidative conditions, *MSN2/4* and *SKN7* are located within the fuzzy zone of the −1 nucleosome (Figure 3C). In contrast, the *DhCTT* promoter contains motifs for *SKN7*, *SKO1*, and *YAP1* (Figure 4C), and no nucleosomes were found in this region, suggesting an open chromatin (Figure 3D).

To gain a deeper understanding of the transcription factors involved in the regulation of oxidative stress in *D. hansenii*, we investigated the presence of shared motifs in the promoters of other genes (*DhSOD1*, *DhENA1*, *DhSTL1,* and *DhGPD1*) that have been experimentally shown to be co-regulated with catalases genes in response to oxidative- or osmotic-stress response [12,35,54]. A motif analysis was performed using Regulatory Sequence Analysis Tools and the position weight matrices from the Jasper database (Figure 4A). As shown in Figure 4D,E, the *MSN2/4-*binding sequences were present in five promoters analyzed, except for *DhCTT*. The motif for the *SNK7* was identified in all promoters. The *SKO1-*binding sequence was found in *DhCTA*, *DhCTT*, *DhSOD1*, and *DhSTL1*. Finally, the *YAP1-*binding sequence was identified in the promoters of *DhCTA*, *DhCTT*, *DhSOD1*, *DhENA1*, and *DhSTL1*. Notably, the most frequently repeated consensus sequence in the *DhCTA* promoter is the *STREs* (*STress Responsive Elements,* NGGGG, or CCCCN), recognized by Msn2/4 for the general stress response (Figure 4B,D,E). In contrast, the *MSN2/4*-binding site is absent in the *DhCTT* promoter (Figure 4C), suggesting that *DhCTT* expression is not transcriptionally activated via Msn2/4.

In addition, to explore the potential conservation of function and regulation of the *Dh*Msn2/4, *Dh*Skn7, *Dh*Sko1, and *Dh*Yap1 proteins in *D. hansenii*, their sequences were compared with the homologous of *S. cerevisiae* and *C. albicans* using Clustal Omega (Figures S3–S6, Table S1). The sequence comparison reveals conserved or semi-conserved residues within critical domains of these proteins (Table 2), suggesting that their functions and regulatory mechanisms are likely conserved among these yeast species. Furthermore, putative binding motifs for Msn2/4, Skn7, Sko1, or Yap1 have been identified in NFRs of the promoter regions of the *DhCTA* and *DhCTT* genes in *D. hansenii*. This analysis reinforces the idea that the function and regulatory mechanisms of these TF are conserved in *S. cerevisiae*, *D. hansenii*, and *Candida albicans*.

### 3.3. DhHog1 Regulates the Activity and Expression of Catalase A Under Saline and Oxidative Conditions

The most conserved stress response signaling cascade in fungi is the HOG pathway, in which the final effector is the MAP kinase Hog1 [49]. This kinase activates transcription factors primarily associated with osmotic and oxidative stress in *S. cerevisiae*, such as Msn2/4 [55] and Sko1 [56,57]. The analysis of NuSA and the identification of binding sites suggest that *Dh*Hog1 may be involved in the transcriptional activation of *DhCTA* and *DhCTT* genes, as their promoters contain putative motifs for Msn2/4 and/or Sko1 (Figure 3C,D and Figure 4), which are linked to Hog1 MAP kinase.

**Figure 5:**
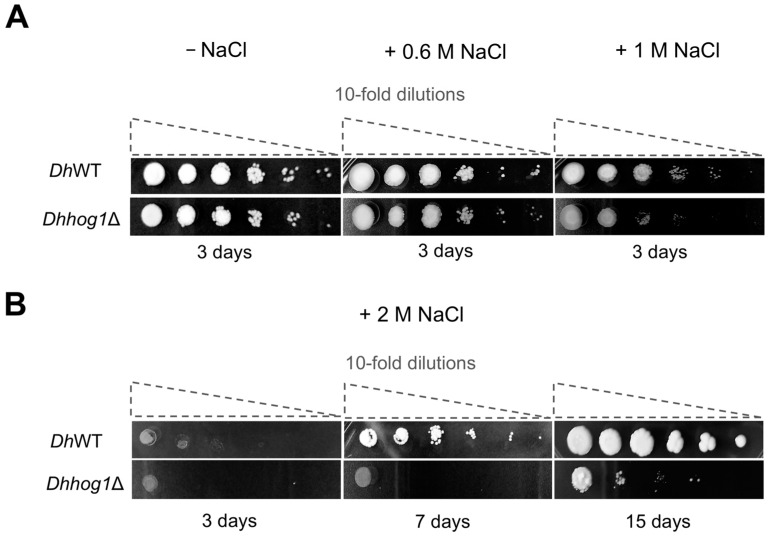
The *Dh*Hog1 MAP kinase is crucial for cellular viability in *D. hansenii* under saline-stress conditions. Cells were grown in rich medium YPD during the mid-log phase, followed by 10-fold serial dilutions (10^−1^, 10^−2^, 10^−3^, 10^−4,^ 10^−5^, 10^−6^). A 10 μL aliquot of each dilution was spotted onto YPD agar medium plates without or with NaCl (+0.6 M, +1 M, or +2 M) and incubated at 28 °C. (**A**) YPD plates without NaCl, with 0.6 M NaCl, or with 1 M NaCl were incubated for 3 days. (**B**) YPD plates with 2 M NaCl incubated for 3, 7, and 15 days. Representative images from three independent experiments are shown.

To evaluate the role of *Dh*Hog1 in oxidative stress response, we performed a series of comparative assays using a *Dhhog1Δ* mutant strain. We measured cell growth and viability in both the WT strain and the *Dhhog1*Δ mutant in rich medium YPD without NaCl and with 0.6 M NaCl (Figure 5A and Figure S7). The results indicate that both strains exhibit similar growth patterns and show no significant differences in cell viability, consistent with previous findings, suggesting that *Dh*Hog1 is not essential at 0.6 M NaCl, although it is active and phosphorylated under these conditions. However, *Dh*Hog1 appears to be crucial for maintaining cell viability under higher NaCl concentrations, such as 1 or 2 M NaCl (Figure 5A,B).

**Figure 6:**
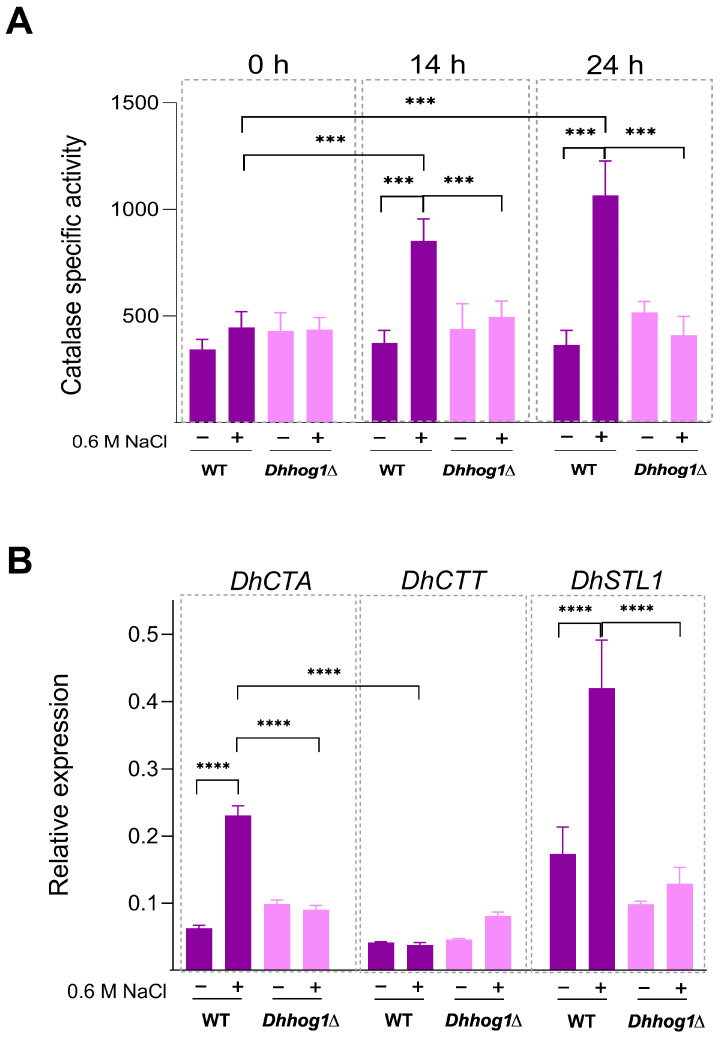
The *Dh*Hog1 MAP kinase regulates the induction of catalase activity corresponding to *DhCTA* expression under NaCl conditions. (**A**) Catalase activity in cell-free yeast extracts was measured from the WT and *Dhhog1*Δ mutant in YPD without (−) or with 0.6 M NaCl (+) at different intervals (0, 14, and 24 h). Bars represent catalase-specific activity (μmol H_2_O_2_ oxidized min^−1^ mg of protein^−1^). (**B**) Total RNA was extracted from WT and *Dhhog1*Δ mutant in YPD without (−) or with 0.6 M NaCl (+) at 14 h and analyzed by RT-qPCR. Bars represent relative expression of the *DhCTA*, *DhCTT*, and *DhSTL1* genes, respectively. Transcript data were normalized against the expression level of the actin gene (*DhACT1*). Values are presented as the mean of at least six independent measurements ± (SD). Significant differences: *p*-value ≤ 0.001 (***), ≤0.0001 (****).

Specific catalase activity was measured in both WT and *Dhhog1*Δ mutant cultured in rich medium YPD and YPD supplemented with 0.6 M NaCl at 0, 14, and 24 h (Figure 6A). As we previously reported [37], the WT strain exhibited an increase in catalase activity under NaCl. However, in a *Dhhog1*Δ mutant, catalase activity did not increase, suggesting that *Dh*Hog1 plays a role in catalase activation under this condition. To further investigate the catalase regulation, we analyzed the transcript levels of *DhCTA* and *DhCTT* genes, using *DhSTL1* expression as a control of osmotic response (Figure 6B). The expression pattern of *DhCTA* mirrored that of *DhSTL1*, which is positively regulated by *Dh*Hog1. This indicates that *Dh*Hog1 mediates the induction of both genes under NaCl conditions. In contrast, *DhCTT* did not show increased transcription in the presence of NaCl, suggesting that *DhCTT* expression is not positively regulated by NaCl or *Dh*Hog1.

**Figure 7:**
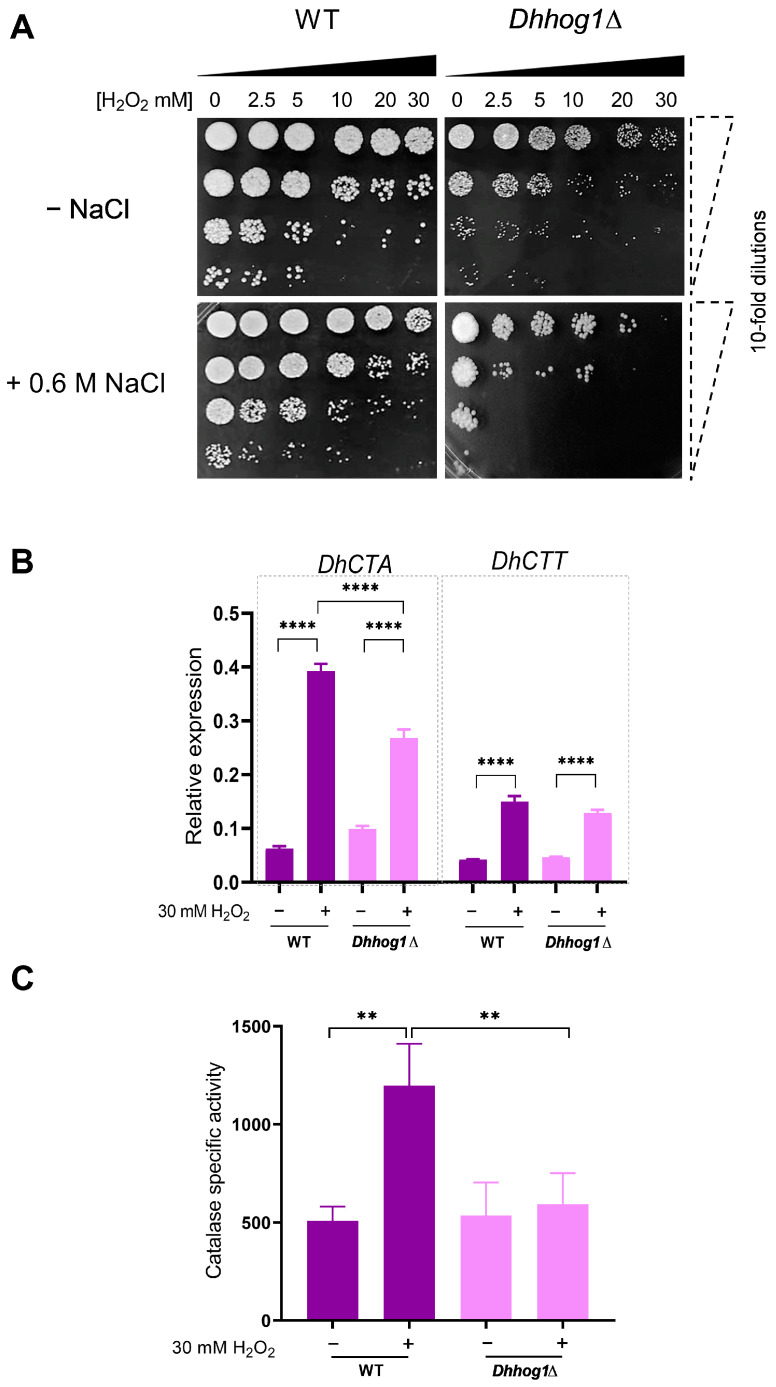
H_2_O_2_ sensitivity and *DhCTA* and *DhCTT* gene expression in WT and *Dhhog1*Δ strains. (**A**) Cells were cultured in YPD without or with NaCl (-NaCl or +0.6 M NaCl). Sensitivity assays with different concentrations of H_2_O_2_ (0, 2.5, 5, 10, 20, and 30 mM) were shocked, followed by 10-fold serial dilutions (10^−1^, 10^−2^, 10^−3^, 10^−4^). A 10 μL aliquot of each dilution was spotted onto YPD agar plates and incubated for 3 days at 28 °C. (**B**) Gene expression analysis of *DhCTA* and *DhCTT* under YPD with a 30 mM H_2_O_2_ shock in WT and *Dhhog1*Δ strains. Total RNA was extracted from WT and *Dhhog1*Δ mutant subjected to YPD with 30 mM H_2_O_2_ shock for 60 min and analyzed by RT-qPCR. Bars represent the relative expression of the *DhCTA* and *DhCTT* genes. Transcript data were normalized against the expression level of the actin gene (*DhACT1*). (**C**) Catalase activity in cell-free yeast extracts was measured from the WT and *Dhhog1*Δ mutant in YPD without (−) or with 30 mM H_2_O_2_ (+) after 60 min. Bars represent catalase-specific activity (μmol H_2_O_2_ oxidized min^−1^ mg of protein^−1^). Values are presented as the mean of at least six independent measurements ± SD. Significant differences: *p*-value ≤ 0.01 (**), ≤0.0001 (****).

To investigate the sensitivity of the *Dhhog1*Δ mutant to oxidative stress under saline conditions, cell viability was analyzed following exposure to varying concentrations of H_2_O_2_ (0, 2.5, 5, 10, 20, and 30 mM) in YPD medium with and without 0.6 M NaCl for both WT and *Dhhog1*Δ strains for 60 min (Figure 7A). The WT strain exhibited sensitivity to H_2_O_2_ at concentrations of 5, 10, 20, and 30 mM, regardless of recovery in YPD. In contrast, the *Dhhog1*Δ mutant displayed heightened sensitivity starting at 2.5 mM H_2_O_2_, with this sensitivity further exacerbated when the shock was performed in the presence of NaCl. The expression analysis of *DhCTA* and *DhCTT* genes following a 30 mM H_2_O_2_ shock revealed that *DhCTA* expression is dependent on *Dh*Hog1, whereas *DhCTT* expression is not influenced by *Dh*Hog1 (Figure 7B). Finally, catalase activity was measured after H_2_O_2_ treatment in both strains, showing a significant reduction in catalase activity in the *Dhhog1*Δ (Figure 7C).

## 4. Discussion

Despite extensive research, the specific mechanisms by which yeasts adapt to extreme environments remain partially understood [58,59]. In the halotolerant yeast *D. hansenii*, catalase A and T play a crucial role in defending against oxidative stress by catalyzing the decomposition of H_2_O_2_ [11,37]. However, the direct interactions between the pathways regulating responses to combined saline and oxidative conditions and the antioxidant defense systems mediated by catalases, superoxide dismutases, thioredoxins, glutathione, and peroxiredoxins remain poorly characterized [20,60,61,62]. Additionally, while the HOG1 pathway is well characterized in *S. cerevisiae* for its role in osmotic stress response, the specific function of *Dh*Hog1 in *D. hansenii* under oxidative stress is still under investigation. Therefore, understanding these mechanisms and adaptations is fundamental not only from an ecological and evolutionary perspective but also has biotechnological implications, as halotolerant yeast can be employed in industrial processes under saline conditions.

**Figure 8:**
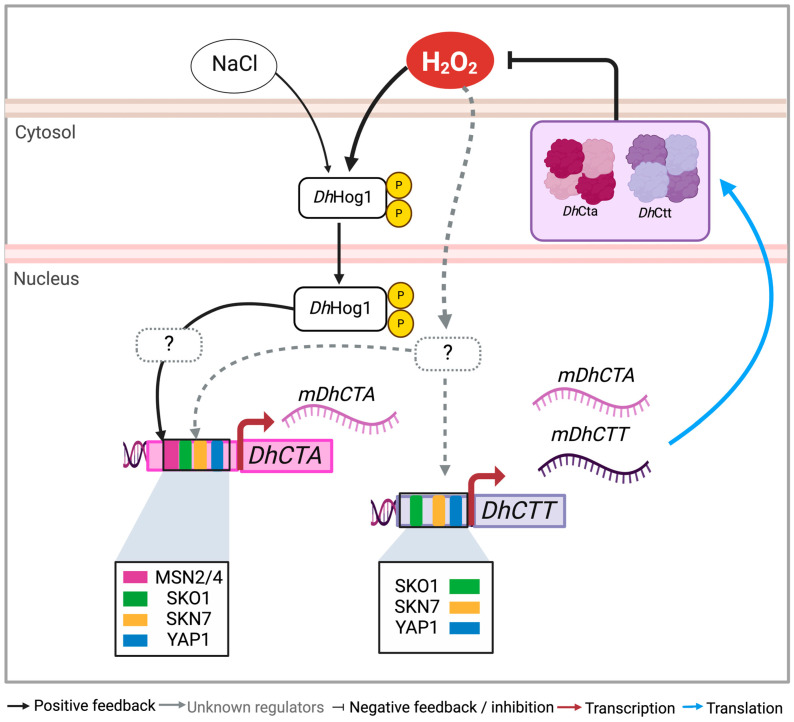
Illustration depicting the positive regulation of *Dh*Hog1 in catalase activity in *D. hansenii* under NaCl and/or H_2_O_2_ conditions. *Dh*Hog1 positively regulates *DhCTA* in both conditions. Binding motifs for stress-related transcription factors Msn2/4, Sko1, Skn7, or Yap1 are located in the promoters of *DhCTA* and *DhCTT* genes. Under oxidative stress, the induction of both genes has an additive effect on catalase transcription and activity to cope with H_2_O_2_ accumulation. Transcriptional factor binding sites are color-coded. Created using BioRender.com with the publication license number TN27DNAJLH.

In this study, we explored the regulatory responses of *D. hansenii* to saline and oxidative conditions (Figure 8). Our findings indicate that under NaCl, an open chromatin state with available binding motifs for Msn2/4, Skn7, Sko1, and Yap1 facilitates the expression of catalases A (peroxisomal/mitochondrial) and T (cytosolic), thereby enhancing the yeast’s capacity to adapt to environmental stress changes. *Dh*Hog1 was identified as a key regulator, specifically controlling *DhCTA* expression under both saline and oxidative conditions, whereas *DhCTT* operates independently of the *Dh*Hog1 function. This suggests a specialized stress response pathway in *D. hansenii* that could be advantageous for industrial applications requiring robust yeast strains.

### 4.1. The Expression of DhCTA and DhCTT Genes Increases After Exposure to H_2_O_2_ Under NaCl Condition

Saline environments present hostile conditions for many fungi due to induced osmotic stress, ionic toxicity, and oxidative stress [63,64]. Such saline conditions significantly hinder the survival of non-adapted species. However, some yeasts have evolved specific mechanisms to resist and thrive in these environments [32,38,65,66]. The case of *D. hansenii* is well documented; this halotolerant yeast has developed adaptations to survive in saline environments, such as efficient osmoregulation, ion transport systems, adapted proteins, and enhanced antioxidant enzyme systems [31,54,67]. Moreover, NaCl has been shown to exert a protective effect against externally triggered oxidative stress in *D. hansenii* [11,68].

In this study, we demonstrated that *D. hansenii* tolerates oxidative stress induced by H_2_O_2_ (30 mM) under typical marine salinity conditions (0.6 M NaCl). After 60 min of exposure, a decrease in cell viability was observed with a transient increase of up to five fold in the expression and activity of catalase *Dh*Cta or *Dh*Ctt, peaking between 60 and 120 min before returning to baseline after three hours.

In the non-halotolerant yeast *S. cerevisiae*, the activity of peroxisomal catalase *Sc*Cta1 remains unchanged, whereas the activity of cytosolic catalase *Sc*Ctt1 significantly increases after exposure to H_2_O_2_. Therefore, *Sc*Cta1 and *Sc*Ctt1 have distinct roles: *Sc*Cta1 primarily clears cytosolic ROS, while *Sc*Ctt1 handles oxidative stress from external sources [69,70,71]. The induction of *Sc*Ctt1 activity is crucial for protecting *S. cerevisiae* against exogenous H_2_O_2_ [18]. In other yeasts, such as *Candida nivariensis*, *Candida albicans*, and *Candida glabrata*, the single catalase enzymes (*Cn*Cat, *Ca*Cat1, and *Cg*Cat1) are upregulated and play significant roles in combating H_2_O_2_-induced oxidative stress [19,39].

Unlike *S. cerevisiae*, our findings demonstrate that both *Dh*Cta and *Dh*Ctt are induced upon H_2_O_2_ exposure in *D. hansenii*, highlighting the essential additive roles of these catalases in mitigating oxidative stress. These results suggest that *D. hansenii* responds to oxidative stress by temporarily upregulating the expression of the *DhCTA* and *DhCTT* genes, indicative of a robust catalase-based defense system against ROS.

As previously described [11], this system maintains moderately high basal catalase activity under saline conditions in *D. hansenii* compared to *S. cerevisiae* (161 ± 5 vs. 8.1 ± 0.5 μmol H_2_O_2_ oxidized min^−1^ mg of protein^−1^). This elevated activity facilitates the simultaneous induction of both catalase A (peroxisomal/mitochondrial) and catalase T (cytoplasmic), thereby enhancing yeast survival under oxidative stress conditions. Further studies are needed to elucidate the individual contribution of each catalase. This will require the construction of single and double knockout strains in *D. hansenii* using advanced genetic engineering techniques such as CRISPR/Cas systems, given the complexity of genetic manipulation in this yeast [54,72,73,74].

### 4.2. Nucleosome Arrangement and Putative TF-Binding Motifs Configurations Between DhCTA and DhCTT Promoters Reveal Divergent Transcriptional Regulation

The transcription start sites are flanked by the first upstream (−1) and downstream (+1) nucleosomes, which are critical regulators of transcription. The +1 nucleosome, noted for its robust position, has been extensively studied due to its role in establishing the structural framework of both repressed and active promoters and its incorporation of histone variants [75,76]. In *S. cerevisiae*, the 5′ nucleosome-free region (NFR) is delineated by −1 and +1 nucleosomes. Specifically, the −1 nucleosome is situated upstream of the NFR, typically within a region ranging from −300 to −150 bp relative to the transcription start sites. Conversely, the +1 nucleosome defines the downstream boundary of the NFR and is, on average, the most strongly localized nucleosome within the yeast genome [77]. In non-conventional yeasts, few studies have investigated the transcriptional regulation between orthologous genes by examining nucleosomal occupancy and putative TF-binding motif configurations within promoter regions [45,78,79].

**Figure 9:**
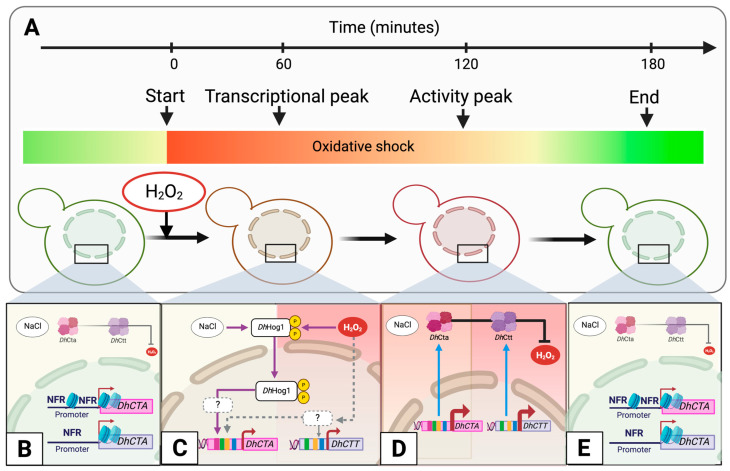
Schematic representation of the regulation of catalase genes *DhCTA* and *DhCTT* in *D. hansenii* during adaptation to oxidative stress induced by H_2_O_2_ shock under saline conditions. (**A**) The timeline of cellular homeostasis (green) and oxidative stress (red) shows the treatment start and peaks in transcription and enzyme activity. (**B**) Promoters of *DhCTA* and *DhCTT* exhibit an open chromatin configuration exhibiting nucleosome-free regions (NFR). (**C**) *DhCTA* expression depends on *Dh*Hog1 under both saline and oxidative conditions, whereas *DhCTT* expression is induced independently of *Dh*Hog1 during oxidative stress. Transcription factor binding motifs related to osmotic or oxidative stress are located in both promoters. (**D**) Catalase activity temporarily increases to handle H_2_O_2_ accumulation. (**E**) Activity returns to basal levels after 180 min of the initial stimulus. Purple arrows indicate positive feedback regulation; gray dashed lines represent unknown interactions; gray-ended lines denote negative feedback regulation; red arrows indicate transcription, and blue arrows indicate translation. Created using BioRender.com with the publication license number QV27DNANS1.

In *D. hansenii*, the role of nucleosome occupancy in relation to specific putative TF-binding motifs involved in gene expression responses to stress conditions has not been explored until now. This study represents the first investigation of nucleosome positioning in *D. hansenii*. Utilizing nucleosome scanning assays, minimal changes in nucleosome positioning were observed for the promoters of *DhCTA* and *DhCTT* under NaCl exposure or combined NaCl with a temporality H_2_O_2_ shock (Figure 9A). This stability is likely due to the growth of cells under saline conditions, which maintain an open chromatin state from the outset, accompanied by a basal level of transcription (Figure 9B). Such an open chromatin configuration may facilitate the induction of both catalase genes in response to environmental stress fluctuations.

Furthermore, the *DhCTA* gene exhibited nucleosomes at positions −2, −1, +1, and +2, whereas the *DhCTT* gene was largely nucleosome-depleted except for the +1 nucleosome, which remained consistently positioned under both conditions. NFRs are typically observed in the promoters of actively regulated genes and/or those containing multiple evolutionarily conserved motifs that recruit TFs [80], which can collaboratively recruit chromatin remodelers to exclude nucleosomes, thereby facilitating access to other target DNA sites. Interestingly, the +1 nucleosome of the *DhCTT* gene exhibits significant differences in micrococcal nuclease protection, indicating that it becomes firmly positioned in cells exposed to H_2_O_2_ shock. We speculated that this firm positioning plays a crucial role in the transcriptional regulation of the *DhCTT* gene. In fact, epigenetic memory of an activated state is preserved on histone variants outside the promoters, such as the +1 nucleosome, while epigenetic memory of a repressed state is maintained by nucleosomes within promoters [76,81,82]. This indicates that a well-positioned +1 nucleosome, in conjunction with the absence of nucleosomes in the promoter region, is essential for sustaining an activated state in the *DhCTT* gene. Future investigations should focus on determining the histone composition or variant turnover of the +1 nucleosome to elucidate their roles in maintaining basal transcription and/or inducing *DhCTT* expression. This will require a Chromatin Immunoprecipitation (ChIP) assay to analyze post-translational modifications of histones.

On the other hand, nucleosome scans allowed us to identify NFRs, which are key to identifying putative TFs-binding motifs in promoter regions for active transcription factors in response to stress conditions. We identified sequences recognized by TFs, such as Skn7, Sko1, and Yap1, in both catalase promoters. However, the sequences of stress response elements (STREs) for Msn2/4 are present only in the *DhCTA* promoter (Figure 8). In *S. cerevisiae*, Yap1- and Skn7-mediated pathways are specifically involved in responses to oxidative and osmotic stresses, respectively [15,20]. Sko1 is a repressor that, when phosphorylated by Hog1, recruits SAGA histone acetylase and SWI/SNF nucleosome-remodeling complexes to activate the expression of stress response genes such as *ScCTT1* [14,83]. Msn2/4 controls the expression of more than twenty genes [84,85], including the *ScCTT1* gene [15]. Osmotic stress induces the recruitment of Msn2/4 and Hog1 to the *ScCTT1* promoter [86]. Our protein sequence comparisons reveal that conserved residues in critical domains of Msn2/4, Snk7, Sko1, and Yap1 suggest similar functions and regulatory mechanisms across yeast species. In our in silico study, we did not find binding motifs for Msn2/4 in the *DhCTT* promoter, whereas the *DhCTA* promoter contains six such motifs. This finding suggests that the regulatory mechanism of *DhCTA* may be similar to that of *ScCTT1*, indicating divergent transcriptional regulation from *DhCTT*.

### 4.3. DhHog1 Regulates the Induction of Catalase A to Adapt Under Saline and Oxidative Conditions

In *S. cerevisiae*, the expression of *ScCTT1* in response to osmotic stress is mediated by the Hog1 pathway through stress response elements (STREs) [87], which are recognized by Msn2/4 [86]. Conversely, H_2_O_2_-induced phosphorylation of Hog1 leads to the phosphorylation of Sko1, resulting in the derepression of *ScCTT1* [57,87]. However, H_2_O_2_ can still induce the expression of *ScCTT1* even in *Schog1*Δ and *Scsko1*Δ mutants, indicating that *Sc*Hog1 and *Sc*Sko1 are not essential for its regulation under oxidative stress [57]. In contrast, the induction of *ScCTT1* in response to osmotic stress is definitively mediated by the Hog1 pathway.

In *C. albicans*, *Ca*Hog1 is essential for the resistance to oxidative stress, as the *Cahog1*Δ mutant exhibits sensitivity to H_2_O_2_ [88]. Moreover, *Ca*Hog1 undergoes activation by phosphorylation when cells are exposed to oxidative stress. Other studies have revealed that *Ca*Hog1 can function as both an activator and repressor [89]. In *C. glabrata*, it has been demonstrated that this yeast can adapt to high levels of H_2_O_2_, and this adaptive response is dependent on *Cg*Yap1/*Cg*Skn7 and partially on *Cg*Msn2/4 [40]. These findings demonstrate that the regulation of *CgCTA1* is primarily controlled by *Cg*Skn7, *Cg*Yap1, and *Cg*Msn4, as the deletion of these TFs renders the cells nearly as sensitive to H_2_O_2_.

In our study, analysis of NuSA and identification of putative TFs-binding motifs suggest that *Dh*Hog1 may be involved in the transcriptional activation of *DhCTA* and *DhCTT* genes in *D. hansenii*, as their promoters contain motifs for *Dh*Msn2/4 and/or *Dh*Sko1 (Figure 9B,C). Under saline conditions, the WT strain exhibited increased catalase activity dependent on *Dh*Hog1, whereas the *Dhhog1*Δ did not, suggesting *Dh*Hog1’s role in catalase activation. *Dh*Hog1 mediates the induction of *DhCTA* but not *DhCTT* under saline conditions. The *Dhhog1*Δ mutant displayed heightened sensitivity to H_2_O_2_, especially under saline conditions, indicating the importance of *Dh*Hog1 in oxidative stress resistance. Expression analysis confirmed that *DhCTA* expression depends on *Dh*Hog1 during saline and oxidative conditions. In contrast, *DhCTT* expression showed no significant changes in the *Dhhog1*Δ mutant under the same conditions.

It is noteworthy that in *S. cerevisiae*, *ScCTT1* is essential for coping with osmotic and oxidative stress. In contrast, in *D. hansenii*, both *DhCTA* and *DhCTT* participate in oxidative stress response, with *DhCTA* being regulated by *Dh*Hog1, similar to the regulation of *ScCTT1* by *Sc*Hog1 in *S. cerevisiae*. We speculate that under non-stress conditions (YPD), *Dh*Sko1 could repress the expression of catalases. In addition, under saline or oxidative conditions, *DhCTA* expression is activated via the *Dh*Hog1-*Dh*Msn2/4 pathway, while *DhCTT* expression may be regulated through *Dh*Yap1/*Dh*Skn7 and/or combining other unknown regulators. Further research is needed to elucidate how these regulatory networks are coordinated under dual stress conditions by different TFs.

## 5. Conclusions

This study explores the mechanisms by which the halotolerant yeast *D. hansenii* adapts to oxidative stress under saline conditions. Upon exposure to H_2_O_2_, the yeast transiently increases the expression and activity of catalase genes *DhCTA* and *DhCTT*. The promoters of these genes exhibit minimal nucleosome occupancy, maintaining an open chromatin state that facilitates rapid transcriptional activation. Binding motifs for stress-responsive transcription factors such as Msn2/4 and Sko1 were identified in the *DhCTA* promoter, suggesting their involvement in its regulation.

Importantly, the MAP kinase *Dh*Hog1 is essential for inducing *DhCTA* expression and boosting catalase activity under both saline and oxidative conditions. In contrast, *DhCTT* expression is independent of *Dh*Hog1, implying divergent regulatory mechanisms from *DhCTA*. These findings highlight the complexity of the stress response pathways in *D. hansenii*, offering valuable insights for engineering more robust yeast strains suitable for industrial applications. This is especially relevant for the use of *D. hansenii* in seawater or saline wastewater by-products from industrial activities, which can serve as feedstock in the transition to green biotechnology.

## Figures and Tables

**Table 1:** Deoxyoligonucleotides used for qPCR analysis.

Gene	Primer	Sequence (5′–3′)	Amplicon Size (bp)
*DhCTA*	*DhCTAFw*	AAGGTCTTGCCACATAAGG	145
	*DhCTARv*	GATCAGCAGAAGCTTCCATG	
*DhCTT*	*DhCTTFw*	ATGAAAGAATTGCTGCTGG	150
	*DhCTTRv*	GGCCAAACTTTCTTAATGGG	
*DhSTL1*	*DhSTL1Fw*	TGGGAATGGCTGACACTTATG	118
	*DhSTL1Rv*	GCTCTTCTACCCAACCTATCAATC	
*DhRPS3*	*DhS3Fw*	AAGGACCCAGCAACCAACAA	148
	*DhS3Rv*	AAGCGGAAGCTTCAACTGGT	
*DhACT1*	*DhACT1Fw*	CCCAGAAGAACACCCAGTTT	125
	*DhACT1Rv*	CGGCTTGGATAGAAACGTAGAA	

**Table 2:** Percentage identity of protein domains in *D. hansenii* compared with *S. cerevisiae* and *C. albicans*. The columns display each domain’s position relative to the first amino acid in *D. hansenii*, the type of domain, and the percentage identity compared to *S. cerevisiae* or *C. albicans*.

Protein in *D. hansenii*	Position	Domain	% Identity		
			*S. cerevisiae*		*C. albicans*
*Dh*Msn2/4	368–419	C2H2 Zinc Finger	75	79	89
*Dh*Skn7	26–131	HSF-type	61		85
	397–513	Response Regulator	73		95
*Dh*Sko1	74–97	Hog1-interaction domain	52		58
	226–372	Repression domain	14		25
	233–243	Hydrophobic patch	63		82
	413–430	PKA interaction region	33		24
	466–487	bZIP	82		91
	491–514	bZIP2	33		75
*Dh*Yap1	39–99	bZIP	62		80
	41–48	NLS	100		100
	243–281	nCRD	44		69
	417–462	cCRD	66		91
	439–457	NES	63		100

HSF = heat shock factor; bZIP = basic leucine zipper; NLS = nuclear localization signal; nCRD = N-terminal cysteine-rich domain; cCRD = C-terminal cysteine-rich domain; NES = nuclear export signal.

## Data Availability

The original contributions presented in the study are included in the article/Supplementary Materials, further inquiries can be directed to the corresponding author.

## Supplementary Materials

The following supporting information can be downloaded at https://www.mdpi.com/article/10.3390/jof10110740/s1, Figure S1. Assessment of cell survival of *D. hansenii* following H_2_O_2_ shock at different concentrations under NaCl condition. Wild-type cells were treated with 0, 10, 20, 30, 40, and 50 mM H_2_O_2_ with shaking for 3 hours, followed by 10-fold serial dilutions (10^−1^, 10^−2^, 10^−3^, 10^−4^, 10^−5^). A 10 μL aliquot of each dilution was spotted onto YPD agar plates and incubated for 3 days at 28 °C. Representative image of three independent experiments. Figure S2. Nucleosome scanning assays in *D. hansenii*. (**A**) Cells were grown in YPD medium with 0.6 M NaCl (0 min) to the mid-log growth phase and treated with 30 mM H_2_O_2_ for 60 min. DNA-proteins cross-linking was performed, and spheroplasts were obtained by zymolyase digestion, followed by MNase digestion at different times, as described in the Materials and Methods section. (**B**) Gel electrophoresis was used to isolate mono-nucleosomal DNA (140–160 bp), as described in Materials and Methods. (**C**,**D**) qPCR analysis was performed to amplify the region of each gene from −625 bp to +250 bp (*DhCTA*) or +280 bp (*DhCTT*) relative to the start of the coding region. The tiled black bars above the scale indicate the DNA fragments amplified by qPCR to examine nucleosome occupancy. (**E**,**F**) A well-positioned nucleosome within the *DhVCX1* coding region (gray oval) was observed in NaCl or NaCl plus 30 mM H_2_O_2_ as the mean starting quantity (MSQ). The relative protection of *the DhCTA or DhCTT region (C,D) was determined* using the peak of the *DhVCX1* region. The red horizontal line represents the amplicon used to normalize. Figure S3. Sequence conservation of Msn2 or Msn4 proteins in *S. cerevisiae*, *D. hansenii*, and *C. albicans*. The amino acid sequences of Msn2/4 from *S. cerevisiae* (*ScMSN2*, ID: NP_013751.1/*ScMSN4*, ID: NP_012861.1), *D. hansenii* (*DhMSN2*, ID: CAG84649.2), and *C. albicans* (*CaMSN4*, ID: XP_723438.2) were aligned using the EMBL-EBI Clustal Omega on 9 July 2024 [50]. In the *S. cerevisiae* sequence, functional features are highlighted: Transcriptional Activation Domain (TAD) (blue) with Motive B (black, underlined), Nuclear Export Signal (NES) (green), and Nuclear Localization Signal (NLS) (yellow). The DNA-binding domain (DBD) (gray) contains the C_2_H_2_ Zinc finger structure with conserved cysteine and histidine residues in red and folding-related sites in blue [90]. Asterisks (*) indicate fully conserved residues; colons (:) indicate semi-conserved residues. Figure S4. Sequence conservation of Skn7 protein in *S. cerevisiae*, *D. hansenii*, and *C. albicans.* The amino acid sequences of Skn7 from *S. cerevisiae* (*ScSKN7*, ID: KZV10951.1), *D. hansenii* (*DhSKN77*, ID: CAG85310.2), and *C. albicans* (*CaSKN7*, ID: AAQ08008.1) were aligned using the EMBL-EBI Clustal Omega on 9 July 2024 [50]. In the *S. cerevisiae* sequence the following features are highlighted: Heat-shock factor type DNA binding domain (HSF-type DBD) (yellow), with F135 and L142 residues, critical for ScSkn7 activity marked in red and helix-3 residues known to be involved in contacting DNA (S137, R140, N143, Y145 and K149) marked in blue; the ROCK1/Kinectin homology region (HR) motif involved in interaction with Rho1 and Mbp1 (pink) and the Response Regulator domain (RR) (gray) with phospho-accepting aspartate residue (D427) [91,92] important for the SLN1-YPD1-SKN7 regulation branch related to the HOG pathway is shaded in green, as well as tyrosine residue (T437) fundamental for interaction with Yap1 is shaded in blue [93,94]. Asterisks represent conserved residues (*), and colons (:) indicate semi-conserved residues. Figure S5. Sequence conservation of Sko1 protein in *S. cerevisiae*, *D. hansenii*, and *C. albicans.* The amino acid sequences of Sko1 from *S. cerevisiae* (*ScSKO1*, ID: NP_014232.1), *D. hansenii* (*DhSKO1*, ID: XP_458864.2), and *C. albicans* (*CaSKO1*, ID: XP_019330633.1) were aligned using the EMBL-EBI Clustal Omega on 9 July 2024 [50]. The *S. cerevisiae* Hog1 phosphorylation site (yellow), with conserved/semi-conserved residues in underlying blue; the PKA phosphorylation site (pink), with conserved residues in boldface; and the DNA-binding domain (blue), with conserved leucine zipper residues in red [56]. Repression-mediating domains (green) show conserved hydrophobic amino acids in purple [95]. Asterisks represent conserved residues (*), and colons (:) indicate semi-conserved residues. Figure S6. Sequence conservation of the Yap1 protein in *S. cerevisiae*, *D. hansenii*, and *C. albicans.* The amino acid sequences of Yap1 from *S. cerevisiae* (*ScYAP1*, ID: KZV08838.1), *D. hansenii* (*DhYAP1*, ID:XP_461648.2), and *C. albicans* (*CaCAP1*, ID: KAL1577880.1) were aligned using the EMBL-EBI Clustal Omega on 9 July 2024 [50]. In the *S. cerevisiae* sequence, the basic leucine zipper domain (bZIP) is highlighted in yellow. Within this domain, the basic region residues that directly interact with base pairs (N74, A77, Q78, F81, and R82) are marked in green, and Yap1-specific residues (Q73, Q78, A80, and F81) are underlined. In the leucine zipper region, the typically hydrophobic residues at positions of the coiled-coil are shown in blue, and conserved leucine residues are marked in red [96,97,98]. The nuclear localization signal (NLS) and nuclear exportation signal (NES) are indicated with black and red boxes, respectively. The two cysteine-rich domains, nCRD and cCRD, are underlined in blue and gray, with conserved cysteine residues highlighted in purple (C303, C310, and C315; C598, C620, and C629, respectively) [99,100,101,102,103]. Asterisks (*) indicate fully conserved residues; colons (:) indicate semi-conserved residues. Figure S7. Growth curves of *D. hansenii* WT and *Dhhog1*Δ mutant without and with NaCl. Cells of WT and *Dhhog1*Δ mutants were cultured in rich medium YPD without NaCl (–NaCl) or YPD with NaCl (+0.6 M NaCl). Growth curves were followed for 72 hours. *n* = 3, and data are the mean ± standard deviation (SD). Table S1. Total percentage identity of each *D. hansenii* protein to their homologs in *S. cerevisiae* and *C. albicans*. Table S2. Deoxyoligonucleotides used for nucleosome scanning assays in *DhCTA* locus. Table S3. Deoxyoligonucleotides used for nucleosome scanning assays in *DhCTT* locus.
