# Supplementary material for: Regulation of Catalase Expression and Activity by DhHog1 in the Halotolerant Yeast Debaryomyces hansenii Under Saline and Oxidative Conditions
Source: J Fungi (Basel). 2024 Oct 26;10(11):740. doi: 10.3390/jof10110740 (PMC11595881; doi:10.3390/jof10110740)
Supplement: Supplementary file 1 [file jof-10-00740-s001.zip › jof-3266780-supplementary.pdf]

Supplementary materials

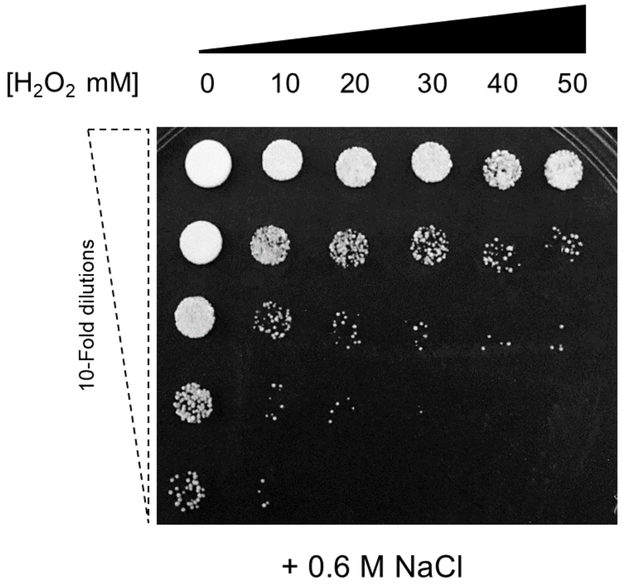

Figure S1. Assessment of cell survival of *D. hansenii* following H_2O_2 shock at different concentrations under NaCl condition. Wild-type cells were treated with 0, 10, 20, 30, 40, and 50 mM H_2O_2 with shaking for 3 hours, followed by tenfold serial dilutions (10^{-1}, 10^{-2}, 10^{-3}, 10^{-4}, 10^{-5}). A10-µL aliquot of each dilution was spotted onto YPD agar plates and incubated for 3 days at 28 °C. Represen tative image of three independent experiments.

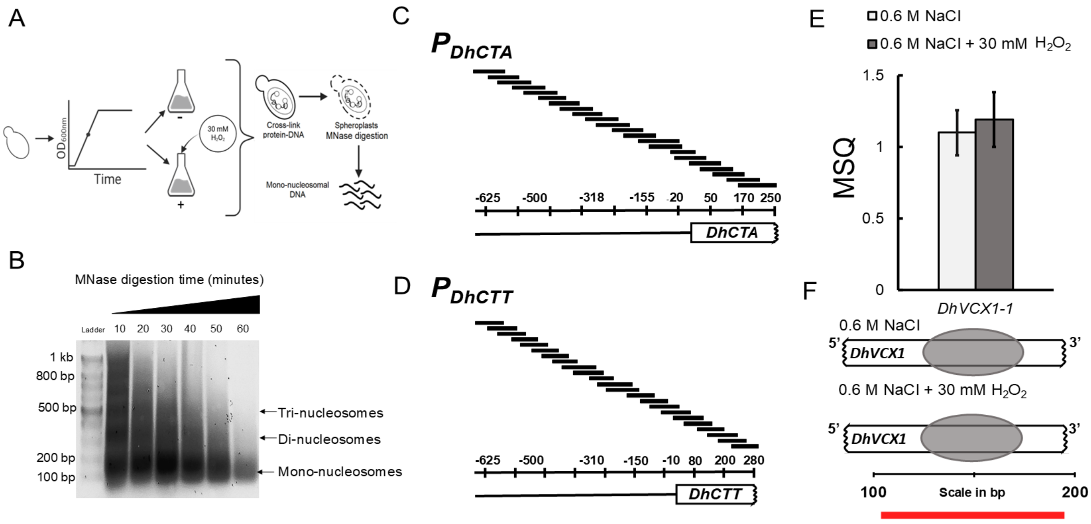

Figure S2. Nucleosome scanning assays in *D. hansenii*. (**A**) Cells were grown in YPD medium with 0.6 M NaCl (0 minutes) to the mid-log growth phase and treated with 30 mM H_2O_2 for 60 minutes. DNA-proteins cross-linking was performed, and spheroplasts were obtained by zymolyase digestion, followed by MNase digestion at different times, as described in the Materials and Methods section. (**B**) Gel electrophoresis was used to isolate mono-nucleosomal DNA (140–160 bp), as described in Materials and Methods. (**C-D**) qPCR analysis was performed to amplify the region of each gene from -625 bp to +250 bp (*DhCTA*) or +280 bp (*DhCTT*) relative to the start of the coding region. The tiled black bars above the scale indicate the DNA fragments amplified by qPCR to examine nucleosome occupancy. (**E-F**) A well-positioned nucleosome within the *DhVCX1* coding region (gray oval) was observed in NaCl or NaCl plus 30 mM H_2O_2, as the mean starting quantity (MSQ).

The relative protection of *DhCTA* or *DhCTT* region (**C-D**) was determined respectively using the peak of the *DhVCX1* region. The red horizontal line represents the amplicon used to normalize.

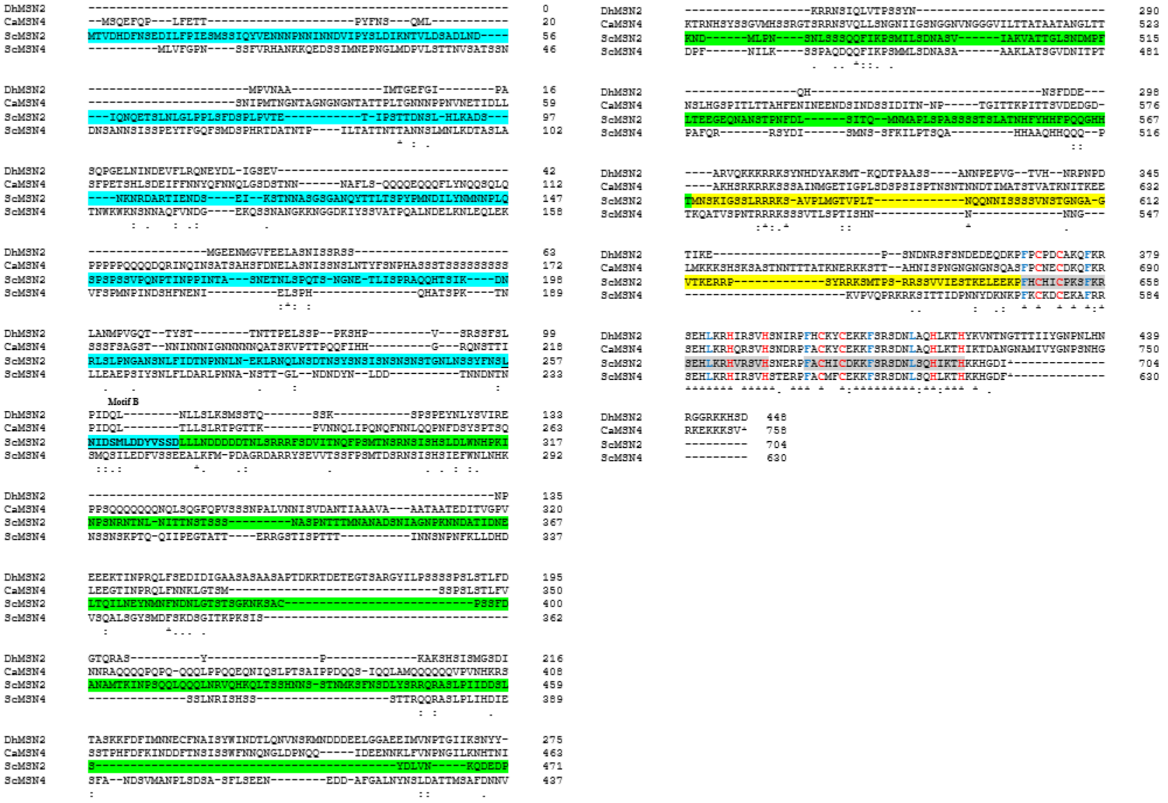

Figure S3. Sequence conservation of Msn2 or Msn4 proteins in *S. cerevisiae*, *D. hansenii*, and *C. albicans*. The amino acid sequences of Msn2/4 from *S. cerevisiae* (*ScMSN2*, ID: NP_013751.1/*ScMSN4*, ID: NP_012861.1), *D. hansenii* (*DhMSN2*, ID: CAG84649.2), and *C. albicans* (*CaMSN4*, ID: XP_723438.2) were aligned using the EMBL-EBI Clustal Omega on 9 July 2024 [50]. In the *S. cerevisiae* sequence, functional features are highlighted, Transcriptional Activation Domain (TAD) (blue) with Motive B (black, underlined), Nuclear Export Signal (NES) (green) and Nuclear Localization Signal (NLS) (yellow). The DNA-binding domain (DBD) (gray) contains the C2H2 Zinc finger structure with conserved cysteine and histidine residues in red and folding-related sites in blue [90]. Asterisks (*) indicate fully conserved residues; colons (:) indicate semi-conserved residues.

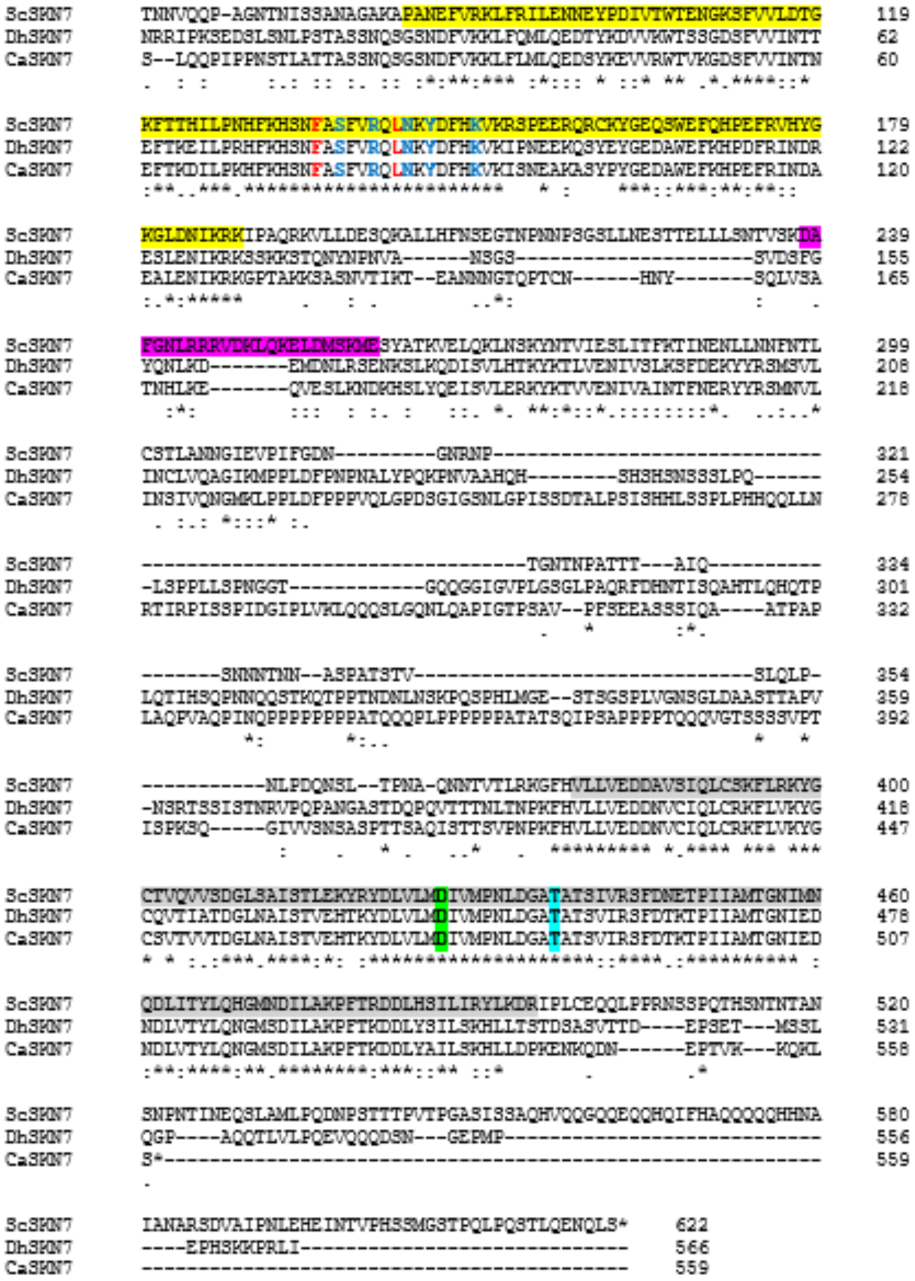

Figure S4. Sequence conservation of Skn7 protein in *S. cerevisiae*, *D. hansenii*, and *C. albicans*. The amino acid sequences of Skn7 from *S. cerevisiae* (*ScSKN7*, ID: KZV10951.1), *D. hansenii* (*DhSKN77*, ID: CAG85310.2), and *C. albicans* (*CaSKN7*, ID: AAQ08008.1) were aligned using the EMBL-EBI Clustal Omega on 9 July 2024 [50]. In the *S. cerevisiae* sequence the following features are highlighted: Heat-shock factor type DNA binding domain (HSF-type DBD) (yellow), with F135 and L142 residues, critical for ScSkn7 activity marked in red and helix-3 residues known to be involved in contacting DNA (S137, R140, N143, Y145 and K149) marked in blue; the ROCK1/Kinectin homology region (HR) motif involved in interaction with Rho1 and Mbp1 (pink) and the Response Regulator domain (RR) (gray) with phospho-accepting aspartate residue (D427) [91,92] important for the SLN1-YPD1-SKN7 regulation branch related to the HOG pathway is shaded in green, as well as tyrosine residue (T437) fundamental for interaction with Yap1 is shaded in blue [93,94]. Asterisks represent conserved residues (*), and colons (:) indicate semi-conserved residues.

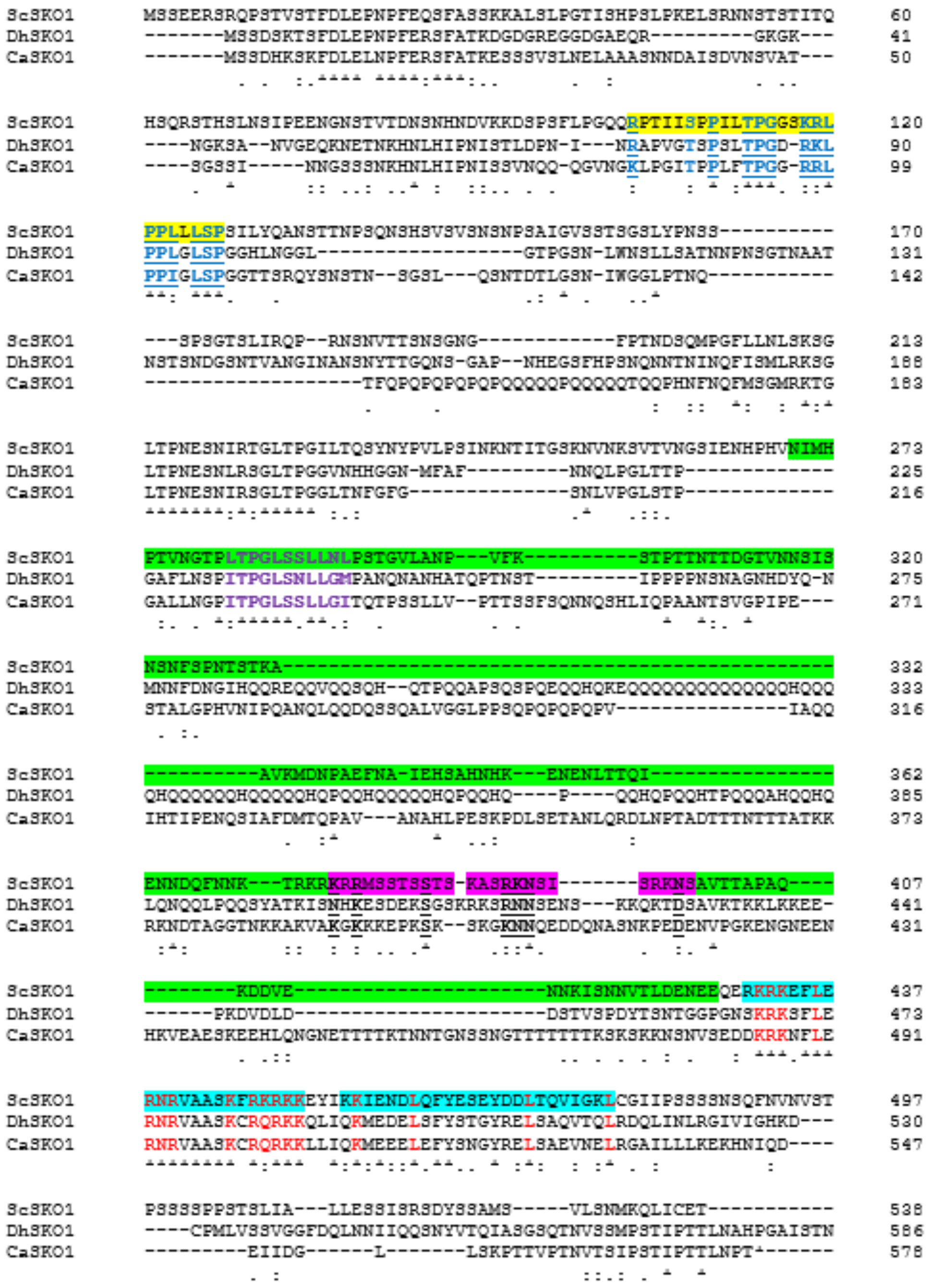

Figure S5. Sequence conservation of Sko1 protein in *S. cerevisiae*, *D. hansenii*, and *C. albicans*. The amino acid sequences of Sko1 from *S. cerevisiae* (*ScSKO1*, ID: NP_014232.1), *D. hansenii* (*DhSKO1*, ID: XP_458864.2), and *C. albicans* (*CaSKO1*, ID: XP_019330633.1) were aligned using the EMBL-EBI Clustal Omega on 9 July 2024 [50]. The *S. cerevisiae* Hog1 phosphorylation site (yellow), with conserved/semi-conserved residues in underlying blue; the PKA phosphorylation site (pink) with conserved residues in boldface; and the DNA-binding domain (blue), with conserved leucine zipper residues in red [56]. Repression-mediating domains (green) show conserved hydrophobic amino acids in purple [95]. Asterisks represent conserved residues (*), and colons (:) indicate semi-conserved residues.

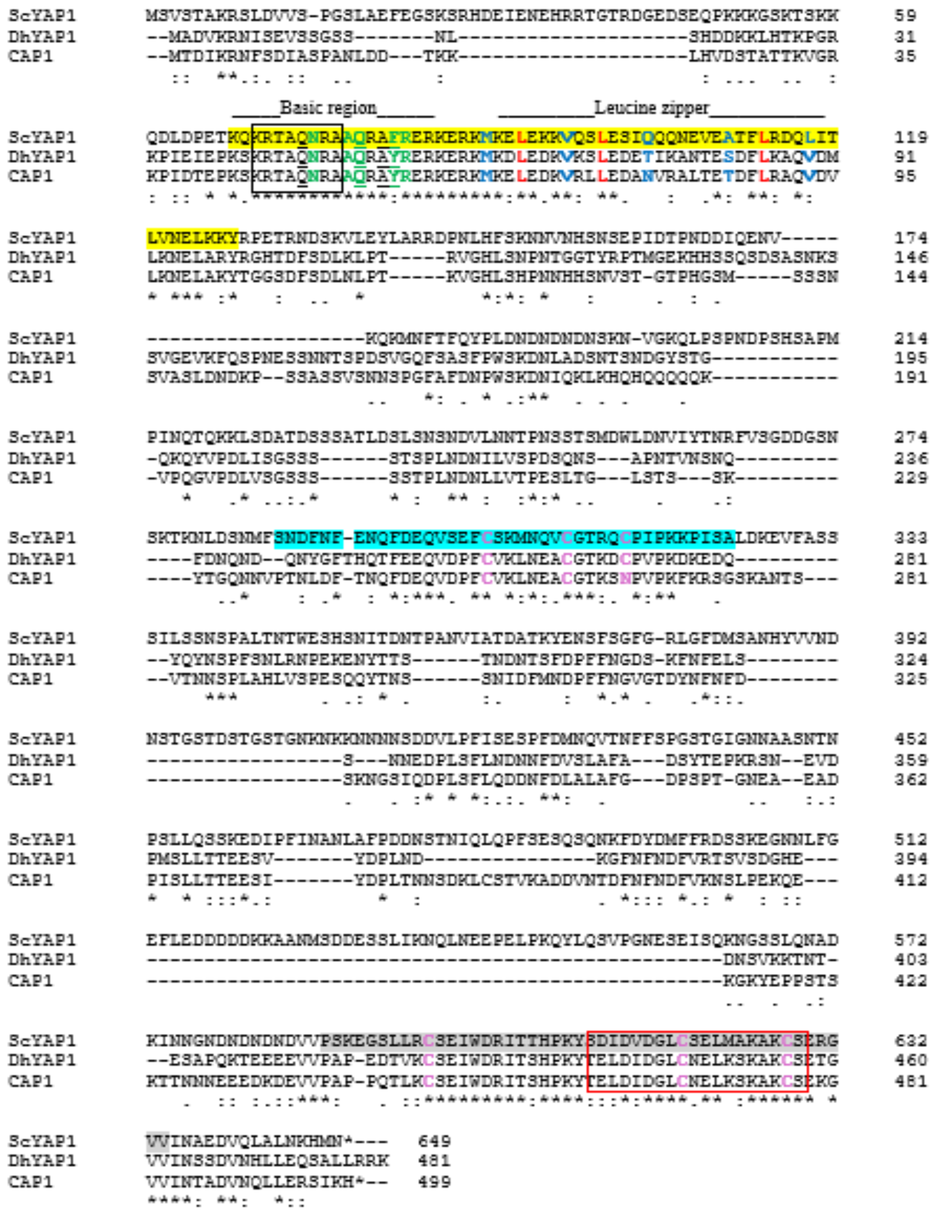

Figure S6. Sequence conservation of the Yap1 protein in *S. cerevisiae*, *D. hansenii*, and *C. albicans*. The amino acid sequences of Yap1 from *S. cerevisiae* (*ScYAP1*, ID: KZV08838.1), *D. hansenii* (*DhYAP1*, ID:XP_461648.2), and *C. albicans* (*CaCAP1*, ID: KAL1577880.1) were aligned using the EMBL-EBI Clustal Omega on 09/07/2024 [50]. In the *S. cerevisiae* sequence, the basic leucine zipper domain (bZIP) is highlighted in yellow. Within this domain, the basic region residues that directly interact with base pairs (N74, A77, Q78, F81 and R82) are marked in green, and Yap1-specific residues (Q73, Q78, A80, and F81) are underlined. In the leucine zipper region, the typically hydrophobic residues at positions of the coiled coil are shown in blue, and conserved leucine residues are marked in red [96–98]. The nuclear localization signal (NLS) and nuclear exportation signal (NES) are indicated with black and red boxes, respectively. The two cysteine-rich domains, nCRD and cCRD, are under-lined in blue and gray, with conserved cysteine residues highlighted in purple (C303, C310 and C315; C598, C620 and C629, respectively) [99–103]. Asterisk (*) indicate fully conserved residues; colons (:) indicate semi-conserved residues.

Table S1. Total percentage identity of each *D. hansenii* protein to their homologues in *S. cerevisiae* and *C. albicans*.

Protein in *D. hansenii*	Total % identity			
	S. cerevisiae		***C. albicans***	
*Dh*Msn2/4	*Sc*Msn2	23	*Ca*Msn2	26
	*Sc*Msn4	27		
*Dh*Skn7	*Sc*Skn7	35	*Ca*Skn7	54
*Dh*Sko1	*Sc*Sko1	22	*Ca*Sko1	35
*Dh*Yap1	*Sc*Yap1	28	*Ca*Cap1	53

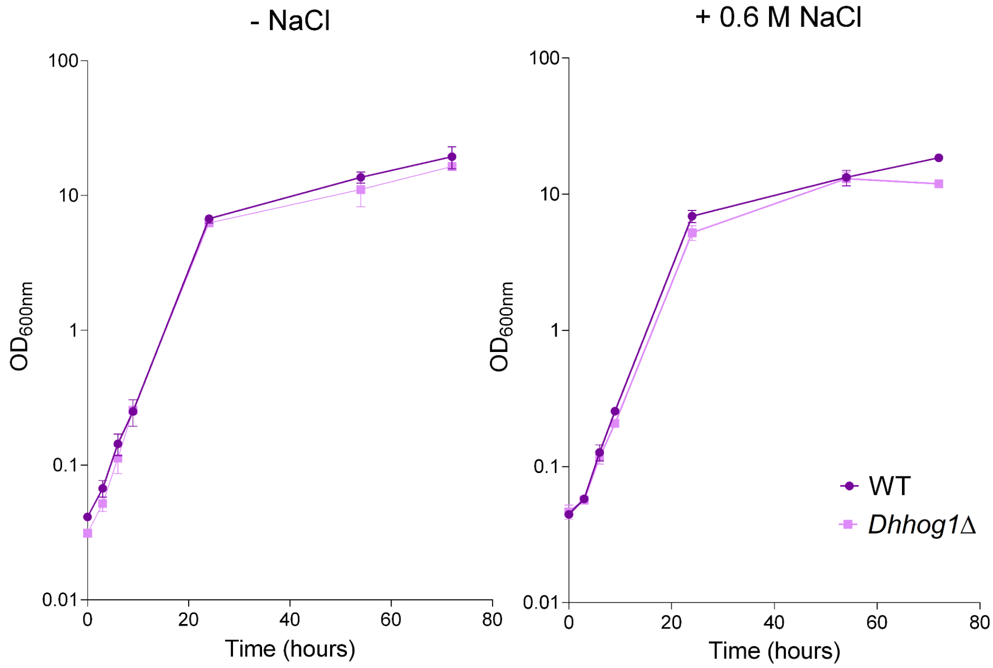

Figure S7. Growth curves of *D. hansenii* WT and *Dhhog1Δ* mutant without and with NaCl. Cells of WT and *Dhhog1Δ* mutant were cultured in rich medium YPD without NaCl (- NaCl) or YPD with NaCl (+ 0.6 M NaCl). Growth curves were followed for 72 hours. n = 3, and data are the mean ± standard deviation (SD).

Table S2. Deoxyoligonucleotides used for nucleosome scanning assays in *DhCTA* locus.

Primer	Sequence (5' – 3')	Middle of amplicon (Promoter coordinate)	5'/3' end	Size (bp)
DhCTA1Fw	TCAGACGGCCAGCCC	-625.5	-676	101
DhCTA1Rv	AGATCAGGAGAAGTTTTACAAGTA		-575	
DhCTA2Fw	CTAATTTAAATGTCTACGAATATCAC	-582	-631	98
DhCTA2Rv	GCTTGAGAAAGAGGTTAGAATATA		-533	
DhCTA3Fw	TACTTGTAAAACTTCTCCTGATCT	-547.5	-599	103
DhCTA3Rv	GACCACCATCGGTACAGATT		-496	
DhCTA4Fw	TATATTCTAACCTCTTTCTCAAGC	-503.5	-557	107
DhCTA4Rv	TTGTGTCAACCCGATATGGC		-450	
DhCTA5Fw	AATCTGTACCGATGGTGGTC	-467.5	-516	97

DhCTA5.Rv	CTCCACAATCTATCAGAGCG		-419	
DhCTA6Fw	GCCATATCGGGTTGACACAA	-428.5	-470	83
DhCTA6Rv	GATGAGGCCATGTGAAGAAC		-387	
DhCTA7Fw	CGCTCTGATAGATTGTGGAG	-391	-439	96
DhCTA7Rv	CTTTGAAGTGTGGACTATCTAG		-344	
DhCTA8Fw	GTTCTTCACATGGCCTCATC	-355	-407	104
DhCTA8Rv	TGAGCACCATCAATCAAGAAGT		-303	
DhCTA9Fw	CTAGATAGTCCACACTTCAAAG	-318.5	-366	95
DhCTA9Rv	TTTCTACATCCTACAGGTATGC		-271	
DhCTA10Fw	ACTTCTTGATTGATGGTGCTCA	-273.5	-325	103
DhCTA10Rv	GGGGTTCAGAAAAGCAGAGT		-222	
DhCTA11Fw	GCATACCTGTAGGATGTAGAAA	-239.5	-293	107
DhCTA11Rv	TAAGTCGCACTCCAAGCATC		-186	
DhCTA12Fw	ACTCTGCTTTTCTGAACCCC	-192.5	-242	99
DhCTA12Rv	GCTTCTAAGTGGCCAGCG		-143	
DhCTA13Fw	GATGCTTGGAGTGCGACTTA	-154.5	-206	103
DhCTA13Rv	ATTTTAGGTAAGCGGGGTGAA T		-103	
DhCTA14Fw	CGCTGGCCACTTAGAAGC	-96.5	-161	129
DhCTA14Rv	TGATGAGATTGTATGATACCTTTTAA		-32	
DhCTA15Fw	ATTCACCCCGCTTACCTAAAAT	-74.5	-125	101
DhCTA15Rv	CCTGTAATTGATGAGATTGTATGA		-24	
DhCTA16Fw	TTAAAAGGTATCATACAATCTCATCA	-18	-58	80
DhCTA16Rv	AGTTAGTGTAAACAGGAGCCAT		+22	
DhCTA17Fw	TCATACAATCTCATCAATTACAGG	+2.5	-48	101
DhCTA17Rv	GCAAATGGTTCTGGGATTGG		+53	
DhCTA18Fw	ATGGCTCCTGTTTACACTAACT	+50.5	+1	99
DhCTA18Rv	ATCTTGCAATAATAATGGACCATG		+99	
DhCTA19Fw	CCAATCCCAGAACCATTTGC	+86	+34	104
DhCTA19Rv	CTTTCTCTGTCGAAGTGTGC		+137	
DhCTA20Fw	CATGGTCCATTATTATTGCAAGAT	+123	+75	96
DhCTA20Rv	AGATCCCTTGGCGTGCAC		+171	
DhCTA21Fw	GCACACTTCGACAGAGAAAG	+168.5	+117	103

DhCTA21Rv	TACAAACATCACTAATATCGTCAG		+220	
DhCTA22Fw	GTGCACGCCAAGGGATCT	+213	+153	120
DhCTA22Rv	GAATCTGGTTAAAACCTTGGTC		+273	

Table S3. Deoxyoligonucleotides used for nucleosome scanning assays in *DhCTT* locus.

Primer	Sequence (5' – 3')	Middle of amplicon (Promoter coordinate)	5'/3' end	Size (bp)
DhCTT1Fw	GATATGTACGTGTTGGTTAATTGT	-625	-676	102
DhCTT1Rv	CTCACTATATGCATACCAACGA		-574	
DhCTT2Fw	TCACAACACTCCACTAACGTAT	-580	-632	105
DhCTT2Rv	CGGAACGAAGTCCGAATCAA		-527	
DhCTT3Fw	TCGTTGGTATGCATATAGTGAG	-546	-596	101
DhCTT3Rv	ACGATCATTCAATCATACGAAGTT		-495	
DhCTT4Fw	TTGATTCGGACTTCGTTCCG	-500	-547	95
DhCTT4Rv	GCTTAACAGCAACTCAAATATTGT		-452	
DhCTT5Fw	AACTTCGTATGATTGAATGATCGT	-467	-519	104
DhCTT5Rv	AGTGGATGTGTAATATAAATGACG		-415	
DhCTT6Fw	ACAATATTTGAGTTGCTGTTAAGC	-429	-476	95
DhCTT6Rv	CTTAACTGGAAGCTTTGTTTGC		-381	
DhCTT7Fw	CGTCATTTATATTACACATCCACT	-393	-439	93
DhCTT7Rv	CGTGAATTCAACGTCAAGATAC		-346	
DhCTT8Fw	GCAAACAAAGCTTCCAGTTAAG	-360	-403	87
DhCTT8Rv	ATATTCAAGAGTGTTGTGGGTC		-316	
DhCTT9Fw	GTATCTTGACGTTGAATTCACG	-309	-368	119
DhCTT9Rv	CGACGAATTACTATACTTTGAACT		-267	
DhCTT10Fw	ACACTCTTGAATATGTTCATTTCC	-276	-330	109
DhCTT10Rv	TCGATTGTTGCTATTGGCTCAA		-221	
DhCTT11Fw	AGTTCAAAGTATAGTAATTCGTCG	-230	-273	87

DhCTT11Rv	AAATTTTTCTGAAGTTGAATACATGATA		-186	
DhCTT12Fw	TTGAGCCAATAGCAACAATCGA	-181	-243	125
DhCTT12Rv	GAACATCGCCTATATTAGTCAG		-118	
DhCTT13Fw	TATCATGTATTCAACTTCAGAAAAATTT	-156	-214	117
DhCTT13Rv	GCAAGCTATGTCTCACTTTCTA		-97	
DhCTT14Fw	CTGACTAATATAGGCGATGTTC	-91	-141	100
DhCTT14Rv	AATGTGTGCTAGGCAGTCGT		-41	
DhCTT15Fw	TTGAGTTAGTTGCTATAATGAAGG	-44	-93	99
DhCTT15Rv	AACCATTTTTACAATAAGCAGTTAGA		+6	
DhCTT16Fw	ACGACTGCCTAGCACACATT	-9	-62	106
DhCTT16Rv	CACTGCTGGATCTTTCTTCG		+44	
DhCTT17Fw	TCTAACTGCTTATTGTAAAAATGGTT	+32	-20	105
DhCTT17Rv	ATGGATGATTGGCATAAGGAAC		+85	
DhCTT18Fw	CGAAGAAAGATCCAGCAGTG	+78	+26	104
DhCTT18Rv	TTGCAATAATAGTGGCCCGC		+130	
DhCTT19Fw	GTTCCTTATGCCAATCATCCAT	+113	+63	100
DhCTT19Rv	CGAAGTGAGAAATGTCGTCC		+163	
DhCTT20Fw	GCGGGCCACTATTATTGCAA	+157	+109	96
DhCTT20Rv	ACCTTTTGCGTGAACAACTCTT		+205	
DhCTT21Fw	GGACGACATTTCTCACTTCG	+198	+143	111
DhCTT21Rv	GTTAAATCAGACAATGAATCAGTC		+254	
DhCTT22Fw	AAGAGTTGTTCACGCAAAAGGT	+232	+181	102
DhCTT22Rv	TAGCCTGGGGATTGTAATGG		+283	
DhCTT23Fw	GACTGATTCATTGTCTGATTTAAC	+279	+230	97
DhCTT23Rv	TTCACCTCCAACTGTAGAGAAT		+327	
